# The *NEWgenerator*^*TM*^ non-sewered sanitation system: Long-term field testing at an informal settlement community in eThekwini municipality, South Africa

**DOI:** 10.1016/j.jenvman.2021.112921

**Published:** 2021-10-15

**Authors:** Hsiang-Yang Shyu, Robert A. Bair, Cynthia J. Castro, Lindelani Xaba, Manuel Delgado-Navarro, Rebecca Sindall, Ruth Cottingham, A. Erkan Uman, Christopher A. Buckley, Daniel H. Yeh

**Affiliations:** a Membrane Biotechnology Lab, University of South Florida, Tampa, FL, USA; b WASH R&D Centre (formerly Pollution Research Group), University of KwaZulu-Natal, Durban, South Africa; c Khanyisa Projects, Durban, South Africa

**Keywords:** Anaerobic membrane bioreactor, Off-grid, Decentralized, Onsite wastewater treatment, Reinvented toilet, MURT, ISO 30500

## Abstract

Globally, there is a dire need for a new class of advanced non-sewered sanitation systems (NSSS) to provide onsite wastewater treatment that is capable of meeting stringent discharge or reuse criteria. These systems need to be simple to operate and maintain, reliable, and resilient to unreliable electrical service. The NEWgenerator (NG) is a compact, automated, solar-powered wastewater treatment system comprised of three major treatment processes: anaerobic membrane bioreactor (AnMBR), nutrient capture system (NCS) with ion exchange and carbon sorption, and electrochlorination (EC). The NG system operated at an informal settlement community in South Africa over a 534 d period, treating high-strength blackwater (BW) and yellow water (YW) from a public toilet facility. Over three test stages (BW, BW + YW, BW) that included several periods of dormancy, the NG system was able to provide a high level of removal of total suspended solids (97.6 ± 3.1%), chemical oxygen demand (94.5 ± 5.0%), turbidity (96.3 ± 9.7%), color (92.0 ± 10.5%), total nitrogen (82.1 ± 24.0%), total phosphorus (43.0 ± 22.1%), *E. coli* (7.4 ± 1.5 LRV, not detected in effluent), and helminth ova (not detected in effluent). The treatment levels met most of the ISO 30500 NSSS standard for liquid effluent and local water reuse criteria. A series of maintenance events were successfully conducted onsite over the 534 d field trial: two membrane cleanings, two NCS regenerations, and granular activated carbon replacement. Desludging, a major pain point for onsite sanitation systems, was unnecessary during the field trial and thereby not performed. The AnMBR performed well, removing 94.5 ± 5.0% of the influent COD across all three stages. The high COD removal rate is attributed to the sub-micron separation provided by the ultrafiltration membrane. The NCS was highly efficient at removing total nitrogen, residual COD and color, but the regeneration process was lengthy and is a topic of ongoing research. The EC provided effective disinfection, but frequent prolonged run cycles due to power supply and water quality issues upstream limited the overall system hydraulic throughput. This extended field trial under actual ambient conditions successfully demonstrated the feasibility of using advanced NSSS to address the global water and sanitation crises.

## Highlights

• A solar-powered onsite sanitation system was field tested in South Africa.
• The unit treated black- and yellow-water at an informal settlement for 534 d.
• Anaerobic membrane bioreactor and nutrient capture system managed carbon and nitrogen.
• Electrochlorination provided disinfection and polishing of effluent.
• ISO 30500 certification readiness and field maintenance requirements were assessed.

## Graphical abstract

**Figure:**
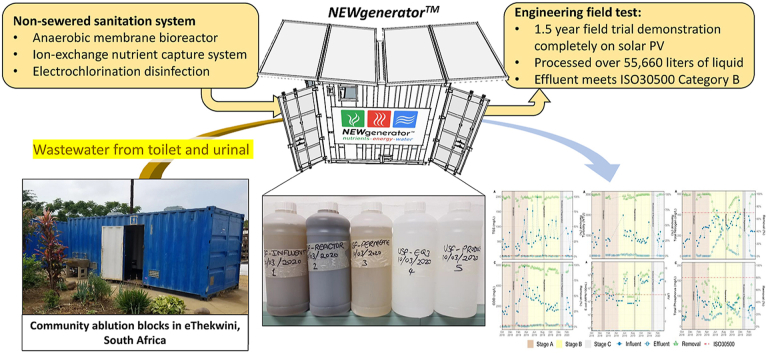

## Abbreviations

ABR: Anaerobic Baffled Reactor
AnMBR: Anaerobic Membrane Bioreactor
BMGF: Bill and Melinda Gates Foundation
BW: Black Water
CAB: Community Ablution Block
COD: Chemical Oxygen Demand
CT: Concentration · Time
d: Days
EC: Electro-chlorination
EFT: Engineering Field Testing
EQ: Equalization
eWS: eThekwini Water and Sanitation
GAC: Granular Activated Carbon
GW: Grey Water
HOCl: Hypochlorous Acid
HRT: Hydraulic Retention Time
ISO: International Organization for Standardization
KP: Khanyisa Projects
LMH: L/m^2^-h
LRV: Log Removal Value
MPN: Most Probable Number
MURT: Multiple-User Reinvented Toilet
NCS: Nutrient Capture System
NG: *NEWgenerator* ^*TM*^
NSSS: Non-Sewered Sanitation System
NTU: Nephelometric Turbidity Unit
OLR: Organic Loading Rate
OP: Omni Processor
ORP: Oxidation Reduction Potential
PVDF: Polyvinylidene Flouride
RT: Reinvented Toilet
RTTC: Reinvent the Toilet Challenge
SURT: Single-User Reinvented Toilet
TMP: Transmembrane Pressure
TN: Total Nitrogen
TP: Total Phosphorous
TSS: Total Suspended Solids
UF: Ultrafiltration
USF: University of South Florida
WRDC: WASH R&D Centre
WW: Wastewater
YW: Yellow Water

## 1 Introduction

Considerable progress in sanitation coverage has been made in the past few decades, however, for many low- and middle-income countries, comprehensive coverage remains out of reach. Approximately 4.2 billion people worldwide still lack access to safely-managed sanitation (WHO, 2019). From a technological perspective, sanitation technologies that can meet the unique treatment needs of a community are critical for providing sustainable long-term sanitation coverage. The most commonly implemented decentralized sanitation technologies in developing communities are pit latrines and septic tanks (Massoud et al., 2009; Graham and Polizzotto, 2013). For high density areas, such as urban slums, high usage rates can render these conventional technologies ineffective or unusable due to pit filling or septic tank overloading. When pit emptying is not coupled with safe fecal sludge management, human waste frequently ends up in open water bodies and becomes an environmental and human health hazard (Strande and Brdjanovic, 2014).

Innovation in the sanitation sector has focused on technological advances for large, centralized WW treatment facilities. These advancements are energy-, chemical- and maintenance-intensive and require significant capital to implement (von Sperling et al., 2001; Diaz et al., 2006). Many communities in developing countries cannot afford the initial capital and long-term operational costs associated with centralized treatment plants and the accompanying sewer systems. Sewer systems are also known to suffer from frequent blockages, leaks, and overflows which can be further damaging to the environment and public health. For such disadvantaged communities, there is a need for advanced decentralized sanitation technologies that can handle the high usage rates, provide high quality treated effluent, generate little to no odor or hazardous byproducts, and accomplish these objectives in a small footprint.

In 2011, the Bill and Melinda Gates Foundation (BMGF) spurred the development of advanced decentralized sanitation technologies by launching the *Reinvent The Toilet Challenge* (RTTC). The aim of the RTTC was to fund research toward the development of a toilet that could treat waste at the point of generation, do so for less than USD 0.05 per user per day, and accomplish it without relying on traditional water and electricity grids (Gates, 2012). From this initiative, several technologies have been developed and field tested, furthering our understanding of human waste treatment strategies. The technologies employed by the reinvented toilets (RTs) include pyrolysis, supercritical oxidation, advanced biological treatment, membrane filtration, electrochemical oxidation, and bioelectrochemical systems. Many technologies have gone from proof-of-concepts to full-scale field tests in developing countries (Bair, 2016; Castro et al., 2014; Cid et al., 2018; Tobias et al., 2017; Sahondo et al., 2020; Varigala et al., 2020). These tests exemplify the versatility of novel approaches in treating human waste while highlighting important technical and social challenges that arise when working in field environments.

In the countries in which RT field tests have been conducted, the regulations related to WW treatment and water recycling were either not well developed or not appropriate for these new technologies. These regulations tend to focus on centralized facilities or decentralized technologies that cannot recycle water (Ragas et al., 2005). Without clearly defined metrics to dictate the performance of an RT, it is difficult to assess whether a new technology is ready for implementation or commercialization. To address this issue, the International Organization for Standardization (ISO) worked with BMGF and other partners to develop an international standard by which to certify the performance and safety of these emerging technologies called non-sewered sanitation systems (NSSS). In 2018, ISO 30500 was introduced, with the aim of establishing performance, safety, reliability, and maintainability standards for NSSS (ISO, 2018).

ISO 30500 specifies WW treatment objectives including meeting effluent water quality criteria such as chemical oxygen demand (COD), total suspended solids (TSS), pH, and key microbial indicators such as *E. coli* and viable helminth ova. The standard also includes nutrient reduction requirements for total nitrogen (TN) and total phosphorous (TP). The standard differentiates between Category A water, which can be used for unrestricted urban uses, and Category B water, which can be discharged or used in restricted urban uses. The standard also distinguishes between three classes of technologies that all fall under the umbrella of an NSSS. Class 1 technologies consist of only one front-end toilet with a back-end treatment system that does not use biological treatment. Class 2 technologies have one front-end unit, but includes biological treatment, while Class 3 systems have more than one front-end toilet connected to one back-end treatment unit. Collectively, these standards provide uniform guidance on the minimum performance and safety requirements for the development and manufacturing of NSSS.

Through BMGF support, the University of South Florida (USF) has developed a compact WW treatment system, called the *NEWgenerator*^*TM*^ (NG). The aim of the NG system is to provide safe sanitation as well as the recovery of nutrients, energy, and water as renewable resources from wastewater. The NG system is a back-end treatment system (Class 3 under ISO 30500) which utilizes anaerobic membrane bioreactor (AnMBR) technology as the core treatment process. AnMBRs combine the advantages of biological anaerobic treatment with the reliability of membrane separation. Membrane separation improves the resiliency of biological treatment by allowing it to handle greater loading fluctuations, reducing the reactor volume, and providing a high-quality effluent (Dereli et al., 2012). The NG system is a fully-integrated system housed in a mini-shipping container which operates entirely on photovoltaic power. The system was previously tested in southern India for one year while treating and recycling WW for toilet flushing at a primary school (Bair, 2016). During that trial, the influent WW strength was relatively low, often under 250 mg/L of COD, whereas typical domestic WW COD ranges from 339 to 1016 mg/L (Tchobanoglous et al., 2003). In that field trial, the NG system was able to remove COD, TSS, and pathogens to meet the local standards. However, nutrient discharge standards were not specified at that time.

This paper presents the performance results from a long-term field trial of an updated version of the NG treating high-strength wastewater generated at an informal settlement community in eThekwini Municipality, KwaZulu-Natal Province, South Africa. During this field trial, the NG system treated two different types of high-strength WW, and the effluent quality was evaluated against local water reuse metrics as well as ISO 30500 standard to determine certification readiness. The objectives of the field trial were to: 1) evaluate the system's ability to treat high-strength WW under a realistic environment, subject to fluctuations in usage and environmental conditions, as well as periods of dormancy, 2) determine system operation and maintenance requirements in such a setting 3) determine potential failure mechanisms and bottlenecks, and 4) identify components, subsystems, and operational procedures that require further modification and optimization to ensure long-term system reliably.

## 2 Materials and methods

### 2.1 System design

**Fig. 1:**
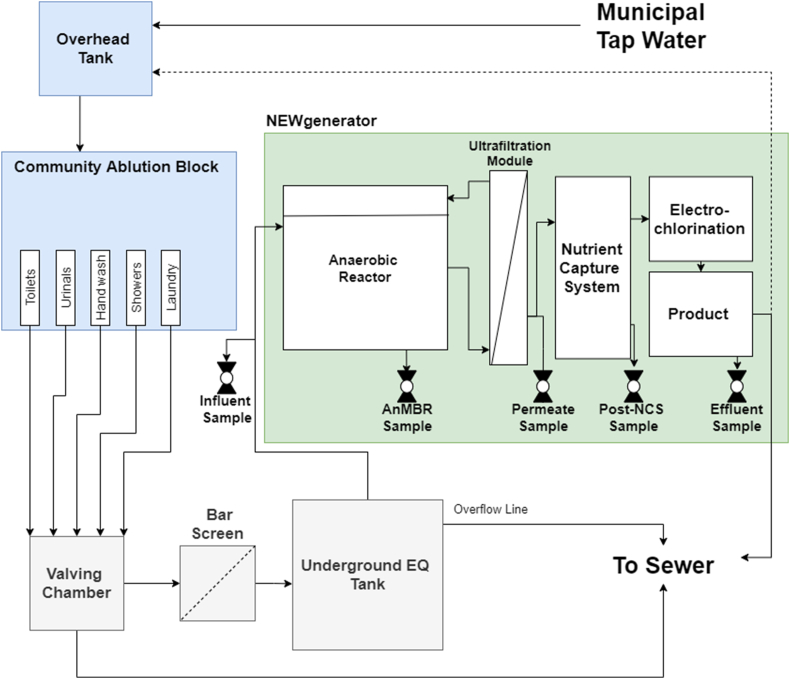
Simplified process flow diagram for the NEWgenerator treatment system and site layout for the engineering field testing (EFT) used in this study. The black valves indicate five sampling locations used for water quality testing. The diagram does not reflect all pumps, valves, sensors, or connectors used. For this study, all effluents were directed to the sewer. Dotted line from product water tank to overhead tank on CAB represents water recycling line for future use.

The NG 100 v.2.0 used in this study was based on a previously field-tested system consisting of an AnMBR and electrochlorination (EC) as the primary treatment processes (Bair, 2016; Bair et al., 2015). However, a key difference in this updated version is the inclusion of a new nutrient capture system (NCS) designed to passively remove nitrogen. The system in this study consisted of three main treatment process steps, including an AnMBR, a NCS comprised of ion-exchange media and activated carbon, and EC (Fig. 1).

**Process Step 1 (AnMBR).** The AnMBR consisted of a three-chamber anaerobic baffled reactor (ABR) with a working liquid volume of 1360 L and a headspace of 160 L. The first two chambers had a coarse polypropylene mesh material which was suspended 1.5 m above the chamber floor to support attached microbial growth. The reactor was designed to operate under ambient temperatures and treat up to 1000 L/day. The last chamber of the reactor was coupled to an external tubular ultrafiltration (UF) membrane module with 15 m^2^ of surface area (X-Flow/Pentair, Minneapolis, MN, USA). The active layer of the UF membrane was made of polyvinylidene fluoride (PVDF) with an average pore size of 0.03 μm. The membrane feed was taken from the midpoint of the last chamber, while the concentrate was returned to the top of the same chamber. As the NG is intended for automated onsite sanitation, the AnMBR was designed for long-term operation with minimal manual maintenance. The usual strategy of high flux, often employed by industrial membrane systems, would result in rapid fouling, a high energy demand for fouling control, and frequent chemical cleaning. Accordingly, the membrane was designed to operate with a low cross flow velocity of 0.1 m/s and a flux of 5 L/m^2^-h (LMH), which were determined to be sustainable over long periods from previous work (Bair, 2016). The membrane operated intermittently according to the water production demands of the system. In between filtration cycles, the membrane was relaxed and backwashed automatically. No additional fouling control measures were utilized. The transmembrane pressure (TMP) was allowed to fluctuate but was monitored closely for any indication of severe membrane fouling. A TMP approaching 0.75 bar would serve as a trigger for chemical cleaning of the membrane.

**Process Step 2 (NCS).** Permeate produced by the membrane module was pumped into an overhead equalization tank. From the overhead equalization tank, the permeate was introduced into the NCS, which consisted of three 360 L tanks connected in series. The NCS was configured such that incoming water entered the bottom of each tank and flowed upwards and out near the top of the tank. Combined, the first two tanks contained 350 kg of clinoptilolite zeolite with an average grain size between 0.85 and 2 mm. The third NCS tank contained 80 kg of granular activated carbon (GAC), which served as a polishing step.

**Process Step 3 (EC).** Effluent from the NCS entered a final equalization tank, from where it was transferred to a 56 L chlorination contact tank. The chlorination tank was operated as a batch process where chlorine gas, generated on-demand through a chloralkaline cell (M100, WaterStep, Louisville, KY, USA), was dosed upon production into the chlorination tank via a venturi tee (Fig. S1, Supplementary Materials). The only input to the chloralkaline cell was a brine solution prepared from NaCl and distilled water. Chlorine gas produced by the chloroalkaline cell was dissolved into the chlorination tank where it was hydrolyzed to hypochlorous acid (HOCl). The advantage of this configuration is that the chloralkaline cell never contacts the wastewater, thereby minimizing electrode fouling and scaling, which is often observed by EC cells that are submerged directly in the process water. The duration of the chlorination cycle was dictated by the measured oxidation reduction potential (ORP) of the water being processed. After reaching an ORP set point, the chlorinated water was released to a product water storage tank. During the field trial, the product water was mainly discharged to the sewer. However, the intent of future NG deployments is to supply non-potable reuse water for toilet flushing and for other applications such as irrigation water for onsite agriculture.

The entire system was housed in a mini shipping container with an internal footprint of 1.9 m long by 2.4 m wide. The electricity for the system was generated by four solar panels mounted to the roof of the container with a combined rated capacity of 1160 W, and stored in a 14.7 kW-hr battery bank. The components in the system operated on either 12 or 24 V DC. The entire system was automated and controlled by an Arduino microcontroller which operated the pumps and valves in response to level sensors in their respective tanks. Photos of the system can be found in Fig. S2.

### 2.2 Site configuration

The NG system was built at the University of South Florida (USF) in Tampa, FL, USA, then shipped to an informal settlement in eThekwini Municipality, KwaZulu-Natal Province, South Africa for installation and testing (Fig. S3). The community, previously described by Sahondo et al. (2020), has a population of 2427 (458 households) and is serviced by six sets of community ablution blocks (CABs, each set consisting of a block for males and a block for females). Provided by the local municipality (eThekwini Water and Sanitation, eWS), the CABs served as the primary source of water, hygiene, and sanitation needs for the community. The NG system was connected to one of the male CABs. Every male CAB had 3 toilets, 2 urinals, 2 showers, 2 handwash sinks, and 2 laundry basins (Fig. S4). For the CAB utilized for the engineering field testing (EFT), the waste lines were modified such that all the WW sources (i.e., sinks, showers, toilets, urinals, and laundry) could be individually sent to the NG system or bypassed to the sewer, depending on the desired testing protocol (indicated by the Valving Chamber in Fig. 1). Any WW that was not sent to the NG system for treatment was diverted to the sewer. The WW that was sent to the NG system for treatment passed through a 20 mm coarse bar screen for trash removal and then entered a 2.5 m^3^ underground equalization (EQ) tank. The working liquid volume of the EQ tank was 2.0 m^3^, with an estimated residence time of 3.8 d based on an average daily blackwater volume of 530 L/d. A 25-mm intake pipe was installed with an opening located 0.5 m from the bottom of the tank to avoid pipe clogging from solids or floating material. Influent was pumped from the EQ tank into the NG system with a diaphragm pump. Since the field trial was a treatability study focusing on removal efficiency, the NG system was designed to treat only a fraction of the wastewater volume generated by the CAB. Excess WW not treated by the NG system overflowed from the underground EQ tank to the sewer. Photos of the EFT site can be found in Fig. S5.

### 2.3 Startup period

A local prototype engineer was trained to operate and maintain the system full-time. Prior to system startup, locally-sourced zeolite and GAC were rinsed with tap water until no fines were visually detected in the output rinse water, then added to the NCS. The anaerobic reactor was seeded with 150 L of anaerobic sludge obtained from a mesophilic anaerobic digester located at the KwaMashu WW Treatment plant in eThekwini Municipality. During startup, the reactor was operated at approximately 250 L/d, a lower rate than full design capacity, to allow the system's microbial culture to acclimate to the characteristics of the influent wastewater. The reactor was officially started on 30 July 2018. The data from the startup period, which lasted 60 days, was not reported in this study since it included adjustments intentionally made as part of training.

### 2.4 System operation

The system's performance was evaluated and reported from October 2018 to March 2020, for a total period of 534 d (458 d operational and 76 d dormant). For this study, the dormancy time is defined as periods when the NG was not operated for over a week due to scheduled shutdown or maintenance events (Table 1). Two different types of WW generated from the CAB were tested to evaluate the system's treatment performance. The first WW type consisted of toilet water only and was denoted as blackwater (BW), while the second WW type included both toilet water and urinal water and was denoted as blackwater + yellow water (BW + YW). The system was operated in three testing stages, labeled as Stage A, B, and C, to identify the effects that the additional urine had on critical performance parameters such as COD and nutrients. During the entire testing period, the NG system was operated under ambient temperature ranging from 13.8∘C to 34.0∘C without any temperature control (Fig. S6).

**Table 1:**
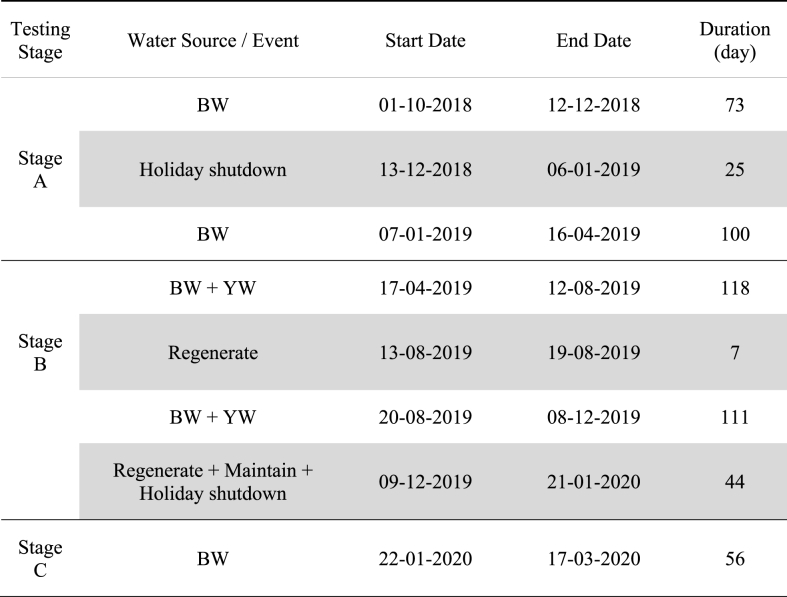
Summary of the field trial timeline with description of key events (BW = blackwater; YW = yellow water; grey box denotes periods offline). Entire field test: 534 d (458 d operational, 76 d dormant); Stage A: 198 (173, 25); Stage B: 280 (229, 51); Stage C: 56 (56, 0). Stage C ended on 17-03-2020 due to general precautions related to COVID-19*.*

#### 2.4.1 Stage A: blackwater treatment

After startup, the system entered Stage A, which consisted of only BW entering the NG system. All other waste streams generated at the CAB were diverted to the sewer. This stage started on 1 October 2018 and ended on 16 April 2019, and lasting 198 d (173 d operational and 25 d dormant). The system was placed on a scheduled shutdown during the summer holiday break in South Africa from mid-December to mid-January. During this period, the prototype engineer was not onsite to operate and maintain the system; therefore, the NG system was dormant. During the 25 d dormancy, the system was shut down without special provisions for maintaining biological activity. All pumps were off, there was no internal recirculation (including membrane loop), and the reactor contents were allowed to settle. Upon startup, the system was immediately returned to service without an adjustment period.

#### 2.4.2 Stage B: blackwater and yellow water treatment

During Stage B, the NG treated an aggregate of BW and YW. This stage started on 17 April 2019 and ended 21 January 2020, for a total of 280 d (229 d operational and 51 d dormant). In addition to the scheduled Dec–Jan holiday shutdown, the NG system was also dormant to conduct two NCS regenerations. The NCS performance and regeneration procedure is thoroughly discussed in Castro et al. (2021). In summary, the onsite regeneration was conducted by isolating the zeolite beds from the NCS and recirculating a regenerant solution of 60–67 g NaCl/L and amended with NaOH to elevate the pH to 10–11. After the regeneration was complete, the zeolite beds were rinsed with tap water then hydraulically reconnected to the NG system. The duration of the regeneration, plus the subsequent rinsing of the bed to remove residual brine, lasted up to 5 d, and the NG system was offline during this period. During Stage B, chemical cleaning of the membrane module was also conducted on 12 November 2019 and 12 December 2019. The membrane was cleaned by running tap water through the membrane loop until the water exiting the membrane loop ran clear. After this rinsing, a cleaning solution of bleach diluted in tap water (500 ppm HOCl) was added to both the feed and concentrate side of the membrane module and soaked for 1 h. The solution was rinsed out of the membrane loop by flushing with tap water for 30 min following the cleaning.

#### 2.4.3 Stage C: return to blackwater

During Stage C, the system again treated BW from 23 January 2020 until 17 March 2020 after which the system was placed on an extended shutdown due to the COVID-19 lockdown implemented by the South African government. The total operational time during this stage was 56 d, with no days dormant.

### 2.5 Sample collection and analysis

Samples were collected from five locations across the system to evaluate the performance of each NG process. These locations included: the influent WW after passing the underground EQ tank (Influent); the last chamber of the ABR which also represented the membrane feed water (Reactor); the membrane permeate prior to entering the overhead equalization tank (Permeate), the NCS effluent in the final equalization tank (Post-NCS), and the final product water after EC (Effluent) (Fig. 1). Samples from each location were collected once a week on approximately the same day and time in sealed containers and transported to the lab in a cooler. All samples were stored in refrigeration and processed within 5 days of sample collection.

The water quality parameters were analyzed by the WASH R&D Centre (WRDC, formerly Pollution Research Group) of the University of Kwazulu-Natal (UKZN) in Durban, South Africa. Raw samples were filtered through a 0.45-μm glass fiber filter to distinguish between soluble and particulate fractions. COD, ammonia, TN, and TP tests were conducted using colorimetric Merck test kits and read with a Merck Spectroquant Probe 300 spectrophotometer. TSS was tested using standard methods, color was analyzed using Hach test kits and a Hach DR900 handheld colorimeter, and turbidity was measured using a 2100Q handheld turbidimeter. The pH was also monitored. In addition to measuring the turbidity in the lab, turbidity was also directly measured in the field immediately after the sample was obtained, to verify that sample storage and transportation did not affect the readings. Bacterial analysis of *E. coli* was performed using Colilert 18/Quanti-Tray 2000 most probable number (MPN) test (IDEXX Laboratories) with a 1 MPN/100 mL detection limit. Helminth ova testing was performed by physical recovery, enumeration, and microscopic inspection (Moodley et al., 2008).

### 2.6 Data logging

A data logger (RX series, Onset Computer Corp., Bourne, MA, USA) was used to remotely monitor the system's performance. The data logger was connected to three pressure transmitters located on the feed, concentrate, and permeate lines of the membrane module. Additionally, several other parameters, including air temperature in the container, liquid temperature in the bioreactor, ORP in the chlorination tank, the total number of batches processed by the chlorinator, and the total volume of water generated in the product tank, were also recorded by the data logger.

## 3 Results

### 3.1 Influent characterization

During Stage A, only BW was directed to the NG system. The BW contained fecal matter, urine, toilet paper or substitutes, flush water, and incidental trash that may be discarded into the toilets. Lacking the diluting influence of greywater, the BW was 5–10 times stronger than typical municipal WW in terms of TSS, COD, turbidity, color, TN, and TP concentrations (Fig. S7). During Stage B, YW captured from the urinals was added to BW. The urinals did not use flush water, therefore, the YW was all urine. Accordingly, the YW contributed additional COD and nutrients to the WW influent with very little to no dilution. Stage C returned to just BW addition, which resulted in influent characteristics similar to those observed in Stage A.

The concentrations for COD, TSS, turbidity, and TP in this high-strength WW did not vary substantially between the three stages, although high variability of those parameters was observed between individual sampling events. An increase in influent constituents was observed during Stage A, which stabilized during Stage B. Some of this increase can be attributed to an accumulation of solids in the underground EQ tank and their subsequent solubilization. The phenomenon was particularly applicable to Stage A, which experienced more holiday and maintenance shutdowns compared to the other stages. Overall, about 50–60% of the total influent COD was in the soluble fraction.

### 3.2 Treatment performance: TSS, COD, turbidity, color, pH

**Fig. 2:**
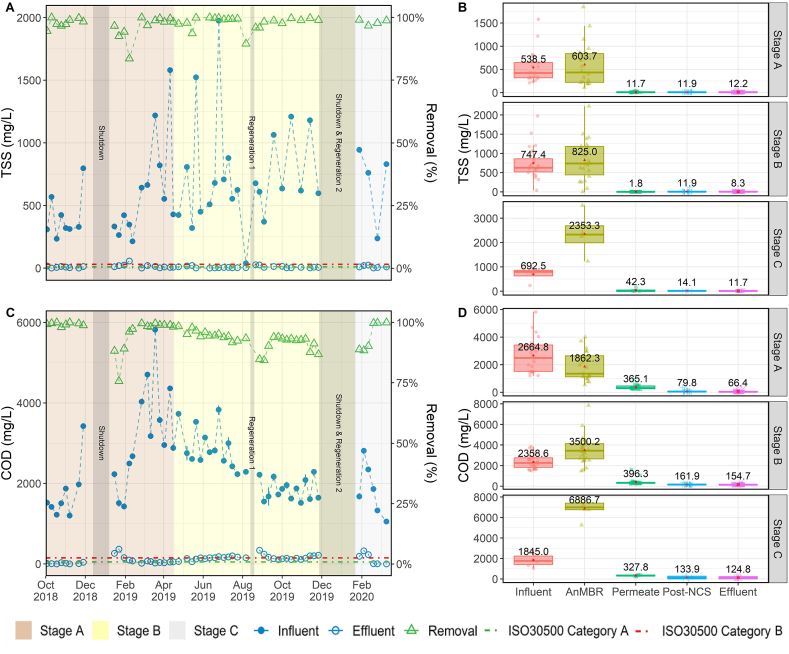
TSS and COD profiles over the three testing stages. Plots A, and C show influent, effluent, and removal percentage of each parameter. The first grey bar shows the timeframe of first shutdown period. Second grey bar shows the timeframe of regeneration of the NCS. The third grey bar shows the timeframe of second shutdown and the second regeneration of NCS. Plots B, and D show the boxplot of each parameter in different steps of the treatment processes. The lines in the box indicate medians, the red dots and the number shown in the box is the average for each parameter, boxes 25th and 75th percentiles. (For interpretation of the references to color in this figure legend, the reader is referred to the Web version of this article.)

**Fig. 3:**
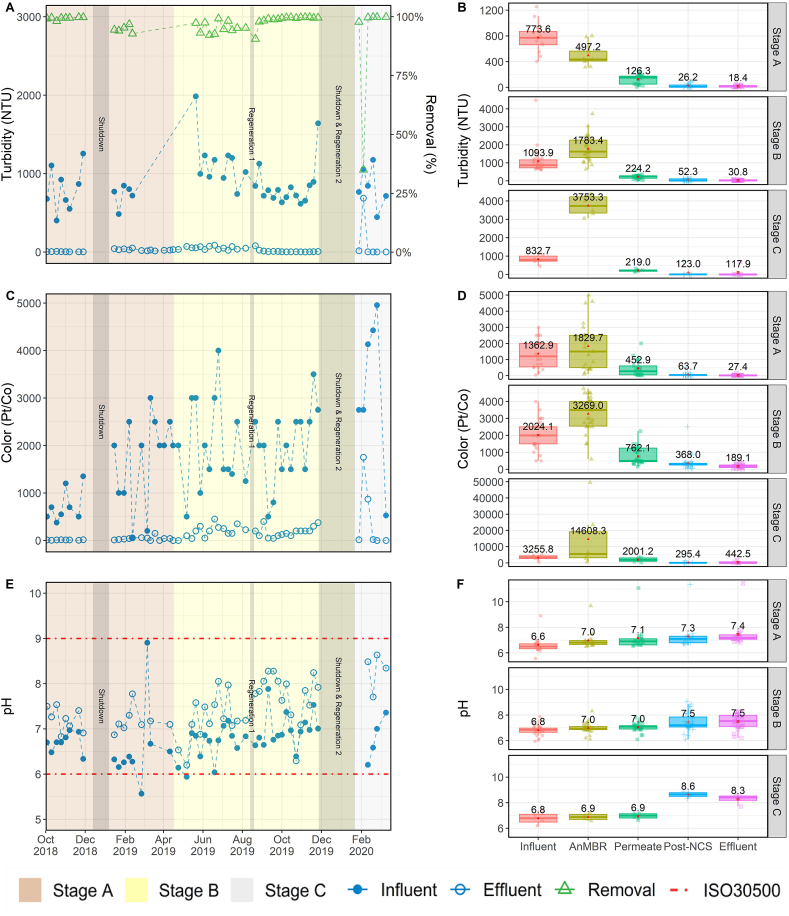
Turbidity, color, and pH profiles over the three testing stages. Plots A, C, and E show influent, effluent, and removal percentage of each parameter. The first grey bar shows the timeframe of first shutdown period. Second grey bar shows the timeframe of regeneration of the NCS. The third grey bar shows the timeframe of second shutdown and the second regeneration of NCS. Plots B, D, and F show the boxplot of each parameter in different steps of the treatment processes. The lines in the box indicate me*dians, the red dots and the number shown in the box is the average for each parameter, boxes* 25th *and* 75th *percentiles.* (For interpretation of the references to color in this figure legend, the reader is referred to the Web version of this article.)

Data for the water quality parameters related to organic matter removal are presented in Figs. 2 and 3. The left column contains *temporal* profiles for influent, effluent and % removal over the three treatment stages, while the right column summarizes *spatial* data at the five sampling locations during each of the three stages. The UF membrane removed a significant percentage of the organics and solids treated by the NG system (Fig. 2 B, D, and Fig. 3 B, D). Membrane filtration was responsible for an average removal of 97.0 ± 9.8% TSS, 82.1 ± 11.9% COD, and 78.1 ± 12.1% turbidity throughout the entire project duration regardless of influent WW type. The membrane served as a reliable barrier to these parameters even under fluctuations of the influent characteristics and the steady accumulation of organic matter in the reactor. The system's ability to retain TSS was due to physical size exclusion by the membrane's sub-micron pores. Accordingly, TSS removal was consistently high and had very little variability throughout the study. Turbidity followed a similar trend, with very little variability in the final product water, apart from a few high values observed in Stage C. These high values were likely the result of chemical precipitation of inorganics caused by oxidation during chlorination rather than issues with the membrane's performance (see Supplementary Materials for expanded discussion).

During the entire 534 d EFT, manual desludging from either the EQ tank or the ABR was unnecessary. The only solids wastage was due to sampling, resulting in a very high solids retention time. Consequently, recalcitrant organic matter gradual accumulated in the AnMBR, as indicated by the gradual increase of TSS, COD, color, and turbidity in the reactor during the field trial (Fig. 2 B, D; Fig. 3 B, D). However, the accumulation in the bioreactor and the corresponding increase of the strength of the membrane feed did not affect the rejection performance of the UF membrane. This was evident by the relatively constant permeate concentrations observed during the field trial. The data suggest that an annual desludging should be sufficient as a scheduled maintenance event to remove accumulated recalcitrant organic matter.

The system's COD removal performance was more complex. During normal operation, the NG was able to remove 94.5 ± 5.0% of the influent COD, decreasing the concentration from 2420 ± 967 mg/L down to an average of 118 ± 91 mg/L. These values do not include data from the adjustment periods following dormancy events. The majority of the COD removal occurred in the AnMBR. In the previous Indian field trial treating dilute WW, the AnMBR alone was able to meet the local COD standard (Bair, 2016). However, in this field trial with higher-strength WW, the permeate COD average was >300 mg/L, requiring additional downstream polishing to meet the ISO30500 COD effluent standard. The GAC in the NCS further removed approximately 200 mg/L of COD from the permeate. Without the addition of GAC, the burden of COD polishing would have been shouldered by EC, which would increase the system's energy demand and run times. During Stage B, the COD removal gradually deteriorated over time, and the product water quality began to increase above the ISO 30500 Category A limit. The increasing effluent COD was attributed to the exhaustion of the GAC bed. Soon after the transition to Stage C, the GAC bed was replaced. Immediately after, the effluent COD returned to below ISO 30500 Category A levels, observing >95% removal. Interestingly, upon restarting the system after the 2018 and 2019 December shutdown periods, the effluent COD values briefly spiked above 300 mg/L. This caused the COD removal efficiency to drop below 80% for several sampling points thereafter (Fig. 2C). It is speculated that the spikes in COD concentration were due to biofilm sloughing off the NCS media immediately following dormancy periods, as the spikes were not observed upstream in the permeate COD.

Color, which is not an ISO 30500 parameter, was also monitored because any observable tint or color in the product water can have an adverse effect on the communities perception and acceptance of water recycling. During the entire period, the NG was able to remove the influent color from 1908 ± 1105 Pt/Co down to 156 ± 267 Pt/Co with an average removal of 92.0 ± 10.5%. Color values followed a similar trend as that of COD (Fig. 3 C, D), with a gradual increase of color in the final product water throughout Stage B and a large spike in color at the beginning of Stage C. The large spike in product color occurred immediately after a long shutdown period. Color in the product water was also significantly reduced to an average of 6.7 ± 11.5 Pt/Co with the GAC replacement. The pH of the process water was observed to increase as it went through the NG (Fig. 3 F). However, the product water pH never exceeded the maximum ISO 30500 value of 9. The average product water pH in the three stages was 7.4 ± 1.1, 7.5 ± 0.6, and 8.3 ± 0.4 (Fig. 3 F), respectively. Over the entire period, the average product water pH was 7.5 ± 0.8 (Fig. 3 E).

Related to the COD removal is the OLR, which indicates the degree of system loading with respect to the limits of biological treatment. The HRT of the AnMBR fluctuated between 1.1 d and 12.4 d (Fig. S8B) in accordance with adjustments made downstream for the EC, or other automation issues. The OLR in the three stages was 0.58 ± 0.59, 0.59 ± 0.39, and 0.39 ± 0.31 g-COD/L-d (Fig. S8A), respectively. During the entire period, the average OLR was 0.57 ± 0.47 g-COD/L-d, while observing periodic spikes as high as 3.81 g-COD/L-d. AnMBRs have been reported to handle an OLR as high as 15 g-COD/L-d before the biological consortia are negatively affected (Shoener et al., 2016). Pilot-scale AnMBRs treating domestic WW typically handle OLR between to 0.4–3 g-COD/L-d (Shin and Bae, 2018). On average, the NG system was operated with an OLR on the lower end of this range, with occasional spikes near the high end. The low OLR of the system indicates that the NG can accommodate additional organic loading before reaching the absolute maximum value of 15 g-COD/L-d. The OLR can be increased by either increasing the hydraulic throughput (lower HRT) or increasing the influent wastewater strength. Increasing the hydraulic throughput can be achieved by either operating the UF membrane at a higher flux (which may not be sustainable from a long-term fouling perspective) or adding additional membrane surface area or module (which increases capital expenditure and operational requirements). An alternate approach is to pre-concentrate the wastewater to increase its organic strength before entering the AnMBR (Dick, 2015). The evaluation of the latter strategy for future NG systems is currently being evaluated and will be the topic of a subsequent publication.

### 3.3 Treatment performance: nutrients

The NCS was designed primarily for TN removal. While incidental TP removal may have occurred due to complexation or precipitation, the current design did not specifically target TP removal. As the NCS was a new feature to this version of NG, the objectives of the EFT for the NCS were to determine the TN removal efficiency and the rate of exhaustion of the beds. The time, chemical, and personnel requirements for onsite regeneration of the NCS were evaluated and described in detail by Castro et al. (2021). The ISO 30500 standard requires that a 70% reduction of TN and 80% of TP be met by the wastewater effluent. The TN and TP concentrations are the only parameters that ISO30500 defines as a percent reduction rather than a specific effluent concentration. As such, the influent concentrations and loading rates of both nutrients are critical parameters in meeting these requirements.

**Fig. 4:**
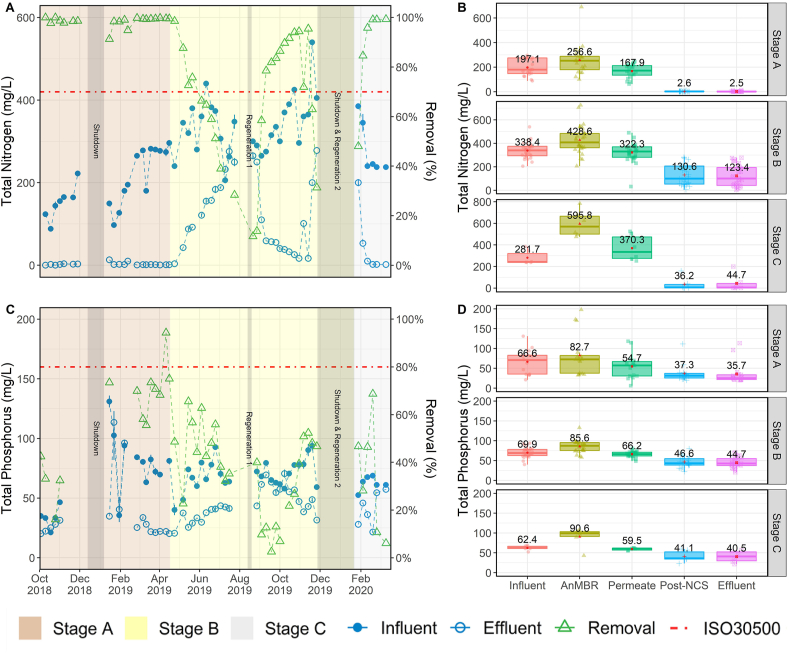
TN and TP profiles over the three testing Stages. Plots A, and C show influent, effluent, and removal percentage of each parameter. The first grey bar shows the timeframe of first shutdown period. Second grey bar shows the timeframe of regeneration of the NCS. The third grey bar shows the timeframe of second shutdown and the second regeneration of NCS. Plots B, and D show the boxplot of each parameter in different steps of the treatment processes. The lines in the box indicate medians, the red dots and the number shown in the box is the average for each parameter, boxes 25th and 75th percentiles. (For interpretation of the references to color in this figure legend, the reader is referred to the Web version of this article.)

During the EFT, the average TN removal efficiency was 98.5 ± 2.1% for Stage A, which met and exceeded the ISO requirement. During Stage B, the influent TN saw a steady increase and fluctuated between 300 and 400 mg/L after the addition of YW (Fig. 4 A). The average influent TN concentrations were approximately 1.8 times higher in Stage B than in Stage A (Fig. 4B). The TN removal rate dropped below the 70% target with an average of 67.6 ± 25.7% as the zeolite beds became exhausted (Fig. 4). In Stage C, the influent TN concentrations dropped once YW was excluded. After the NCS regeneration in Stage C, the target of 70% TN reduction was again met with an average of removal rate of 87.7 ± 20.2%. During the entire EFT, the average TN reduction was 82.1 ± 24.0%, while the NG met the ISO 30500 TN removal standard 67.2% of the time.

Similar to the TN trends, the influent concentration of TP exhibited large increases during Stage A, specifically after the December 2018 shutdown (Fig. 4 C). The influent TP increased to an average of 85.6 ± 83.8 mg/L upon startup in 2019, then dropped to 69.9 ± 12.6 mg/L during Stage B and 62.4 ± 5.9 mg/L in Stage C. The TP removal efficiency in the three stages was 58.7 ± 21.9%, 36.9 ± 18.0%, and 34.4 ± 24.0%, respectively. During the entire period, the average TP removal efficiency was 43.0 ± 22.1% which is below the 80% removal efficiency required by the ISO 30500 standard.

A gradual increase in TN and TP concentrations was observed within the AnMBR over the entire field trial, which followed a similar trend to the increases observed with COD and TSS. This suggests an accumulation of organically-bound nitrogen and phosphorus within the reactor (Fig. 4 B, D). Nonetheless, the membrane was able to decrease the elevated concentrations of TN from 383.5 ± 163.2 mg-N/L in the ABR down to 269.5 ± 111.9 mg-N/L in the membrane permeate. The NCS further decreased the TN concentrations down to as low as 2.6 ± 2.6 mg-N/L in the post-NCS samples during Stage A. The average TN concentration in the post-NCS sample during the whole field trial was 71.6 ± 89.6 mg-N/L. Similarly, the NCS decreased the membrane permeate TP concentration from 68.4 ± 38.0 mg-P/L to 43.0 ± 16.9 mg-P/L in the post-NCS sample. Combined, the AnMBR and NCS treatment steps accounted for the removal of approximately 33.2 ± 54.4 mg-P/L, or about 36% of TP, across all stages.

The NCS effectively removed most of the incoming TN until breakthrough occurred in the NCS zeolite beds after 6 months of operation. This was an indication that the beds had reached saturation. The two zeolite tanks were regenerated twice during Stage B, on Aug 2019 and again on Dec 2019. The regeneration procedure was able to restore the ion exchange capacity of zeolite. After the second regeneration, TN removal efficiency was restored to >90%. The GAC bed was not part of any of the regeneration events, therefore, it was replaced on 18 February 2020 during Stage C. In summary, the performance of the NCS indicates that 1) it can successfully remove TN to very low levels, exceeding the ISO 30500 standard prior to the NCS becoming saturated. 2) the NCS needed to be regenerated after about 6 months of operation, although the frequency may change depending on wastewater strength and cumulative amounts of water processed, 3) the NCS can be regenerated onsite in the field, but the process is lengthy and laborious, and requires further optimization. Further assessment of the NCS regeneration is presented in detail in Castro et al. (2021).

### 3.4 Treatment performance: pathogens

Despite some fluctuations in the nutrient removal efficiencies, the system was highly effective at removing pathogens and reducing indicator species. Throughout the study, low levels of helminth ova (*Ascaris suum*) were detected in the influent, but never in the final effluent (Table 2). The UF membrane was highly effective at removing helminth ova, as the average ova size is between 20 and 80 μm, which is approximately 100 times larger than the average membrane pore size of 0.03 μm (Izdori et al., 2017; Moodley et al., 2008). Due to rejection by the membrane, ova will concentrate within the anaerobic reactor. This concentration strategy allows for the rapid processing of large quantities of WW (10^2^–10^3^ m^3^) over the course of a year, while concentrating solids and pathogens within a relatively small volume (reactor, 10° m^3^) for further treatment at desired times. Most of the ova are expected to become inactivated due to the long residence time in the anaerobic digester (Kato et al., 2003), though some may remain viable. Ultimately, surviving ova can be destroyed by sanitizing the small volumes of concentrated waste sludge (expected to be about 50 L) removed during anticipated annual or biannual maintenance desludging events.

**Table 2:**
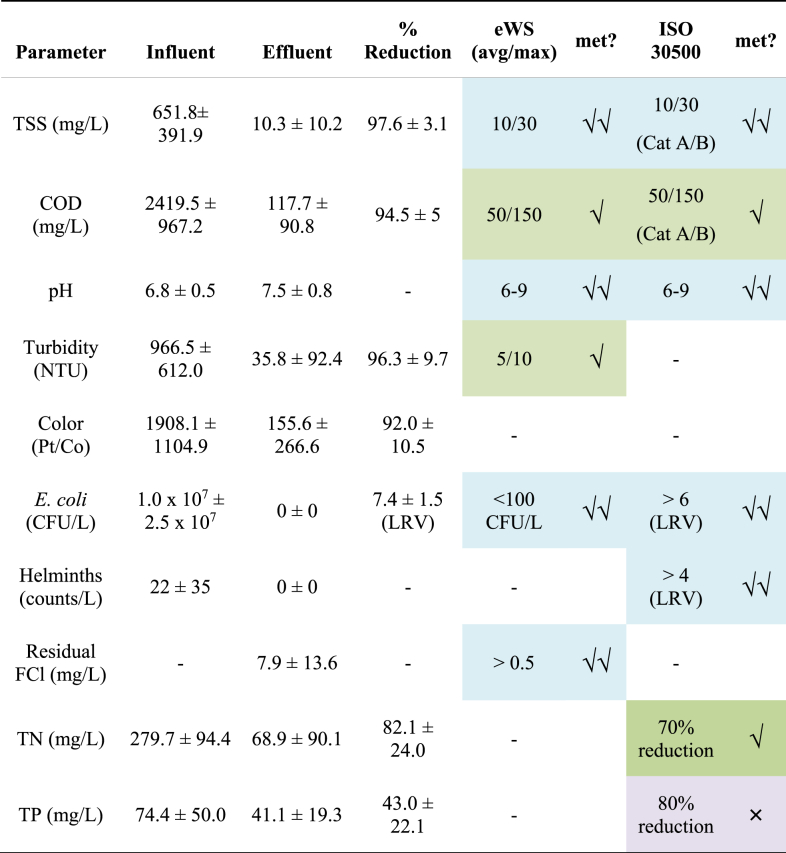
Summary of the system performance over the entire operational period. eWS and ISO 30500 effluent standards are also summarized (shaded blue cell and **√√** indicate standards met by NG for the entire operational period, green cell and **√** indicate standard met for a portion of the operational period, and purple cell and **×** indicate standard not met). Data are mean ± standard deviation of all the measured samples. TSS: total suspended solids, COD: chemical oxygen demand, FCl: free chlorine, TN: total nitrogen, TP: total phosphorus.

**Fig. 5:**
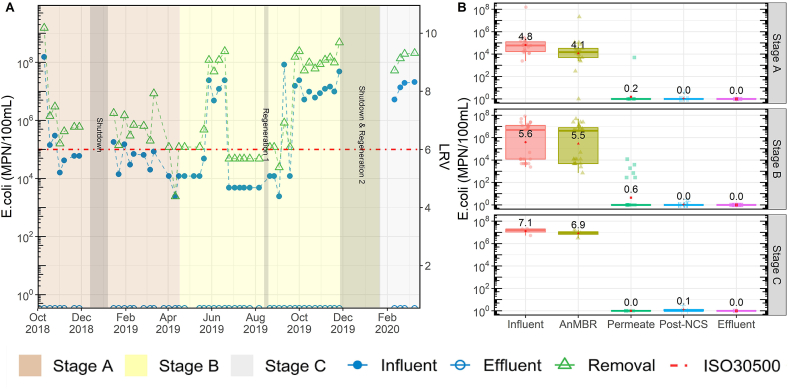
*E. coli* profiles over the three testing Stages. Plot A shows influent, effluent, and log removal value (LRV). The first grey bar shows the timeframe of first shutdown period. Second grey bar shows the timeframe of regeneration of the NCS. The third grey bar shows the timeframe of second shutdown and the second regeneration of NCS. Plot B shows the boxplot of concentration at different steps of the treatment processes. The lines in the box indicate medians, the red dots and the number shown in the box is the average for each parameter, boxes 25th and 75th percentiles. (For interpretation of the references to color in this figure legend, the reader is referred to the Web version of this article.)

The system performed well in terms of the removal of *E. coli* (Fig. 5), with an average log removal value (LRV) of 7.4 ± 1.5. The concentrations of *E. coli* in the influent was 1.0 × 10^7^ ± 2.5 × 10^7^ MPN/100 mL while the effluent concentrations were not detectable (below the 1 MPN/100 mL detection limit). The membrane served as the primary mechanism for *E. coli* removal (Fig. 5B), accounting for 6.7 ± 2.6 LRV of *E. coli* over the entire field trial. Chlorine disinfection served as a second barrier to microbial growth and ensured that the *E. coli* levels were below the detection limit. The EC system was able to produce an average of 7.9 ± 13.6 mg/L of residual free chlorine during the entire field trial, which is higher than the 0.5 mg/L minimum concentration specified in the eWS standard. During the early part of Stage A, the free chlorine in the effluent was significantly higher than the eWS standard, indicating wasted energy and materials with the over chlorination (Fig. S9B). Subsequently, the EC operation was modified to bring down the residual chlorine closer to the standard (Fig. S9A). Free chlorine was not tested during Stage C due to limitations in testing materials at the lab.

There were several weeks from April 2019 to September 2019 during which the ISO 30500 requirement of 6 LRV for *E. coli* appeared to be unmet. However, this was somewhat misleading. The “apparent” LRV was capped at a fixed value due to the way that raw influent samples were measured and reported by the lab. Insufficient sample dilution in the lab caused these influent samples to be reported as a fixed concentration of ≥10^4^ MPN/100 mL. Mathematically, these fixed influent values resulted in a lower-than-expected LRV. The issue was subsequently resolved with a procedural update in the lab. Nonetheless, in no instance was *E. coli* detected in the effluent, thus complying with the other ISO requirement of <100 MPN/L.

It should be noted that in addition to helminth and bacteria, ISO 30500 also includes pathogen removal criteria for protozoa (≥6 LRV) and virus (≥7 LRV). However, it was not possible to evaluate these two pathogen classes during the field trial, as it required surrogate spiking with *Clostridium perfringens* spores (for protozoa) and MS2 Coliphage (for viruses). Surrogate spiking is to be performed in a prescribed manner during the later product certification stage by a manufacturer. However, it is known that UF membrane can physically remove protozoa, while chlorination is effective at inactivating most viruses such as rotavirus, norovirus and hepatitis A at concentration · time (CT) values of 0.05–0.5 mg min/L (CDC, 2012). The CT values produced by NG's EC ranged from 12 to 120 but never less than 2.5 mg min/L. Collectively, the multi-barrier pathogen removal approach undertaken in NG should be effective against these two pathogen classes as well.

### 3.5 Water production

The NG system produced an average of 332 ± 214 L/d of treated effluent during the three stages of operation (Fig. S10), lower than the design rate of 1000 L/d. There are two major considerations related to the production rate: 1) the blackwater influent flow rate was only 530 L/d on the average; 2) EC cycling time controlled the system hydraulic throughput (rather than upstream processes such as membrane flux). Since the EC was operated as a batch process, it often became the rate limiting step for the production of treated water by the NG. The reduced water production was largely the consequence of prolonged EC cycles, ranging from 15 to 50 min, due to a combination of issues with the power supply and post-NCS water quality. During Stage B, it was discovered that a malfunctioning EC power supply was responsible for some of the extended run times. Additionally, the exhaustion of the NCS led to higher levels of chlorine demand in the system water, increasing the amount of time needed for the EC to reach the ORP setpoint of 700 mV. Upon installing a new power supply, the EC system was able to operate at shorter cycle times, occasionally increasing the water production rate by the end of Stage B to near the design rate (Fig. S10). The storage volume of the EQ tank (2 m^3^) enabled the water production to occasionally exceed the blackwater input. Future work should focus on further optimization of the EC cycle, both in terms of operation and hardware, to increase the system's water production rate. Additionally, running simultaneous parallel EC systems or making the EC operation continuous rather than a batch process can further improve the system's overall throughput.

### 3.6 Membrane performance

The membrane performance of the NG system, as indicated by TMP, can be found in Fig. S11. The TMP remained under 0.5 bar during the first half of Stage A but increased to over 0.5 bar during the second half of that stage. This was likely due to minor fouling of the membrane. The TMP remained stable at around 0.5 bar from February 2019 until September of 2019. During that period, there were occasional high TMP values caused by manual increases in the permeate production rate for troubleshooting purposes. Upon returning the flux to the design rate of 5 LMH, the TMP returned to approximately 0.5 bar. After September 2019, the TMP exceeded 0.75 bar, which signaled the need for a chemical cleaning. A chemical cleaning was performed on 12 November 2019, but after only a few days of operation the TMP returned to 0.75 bar until the end of Stage B. A more thorough chemical cleaning, with increased water rinse time prior to bleach cleaning, was conducted before starting Stage C. The additional rinse time removed greater amounts of organic matter deposited on the membrane surface, ensuring that the chemical cleaning focused on harder to remove foulant materials. The increased rinse time successfully reduced the TMP to below 0.5 bar.

## 4 Discussion

### 4.1 Meeting water quality standards

The treatment performance of the NG system over the entire 1.5 yr field trial was evaluated against the metrics set forth by both the ISO 30500 standard and the water reuse standard developed by eWS (summarized in Table 2). The eWS reuse standard mirrored the ISO 30500 requirements for TSS, COD, *E. coli* and pH, but had additional requirements for turbidity and chlorine residual. Nutrients were not part of the eWS reuse standard. The NG system met and exceeded the ISO 30500 requirements for *E. coli* and nitrogen removal. While the average effluent concentrations of COD and TSS did not meet Category A reuse, they did meet the requirements for discharge and category B reuse. Although there are no requirements set by ISO 30500 for turbidity and residual chlorine, eWS requires less than 10 NTU turbidity and a chlorine residual greater than 0.5 mg/L. The NG system was able to meet the chlorine residual, but the effluent turbidity varied across the stages, with an average observed value of 35.8 ± 92.4 NTU. TP was the only parameter that did not meet the ISO 30500 liquid discharge standard of 80% removal. Despite not incorporating a dedicated P removal process step, the updated version of NG system was able to accomplish about half of the ISO requirements, achieving an average TP removal efficiency of 43.0 ± 22.1%.

**Fig. 6:**
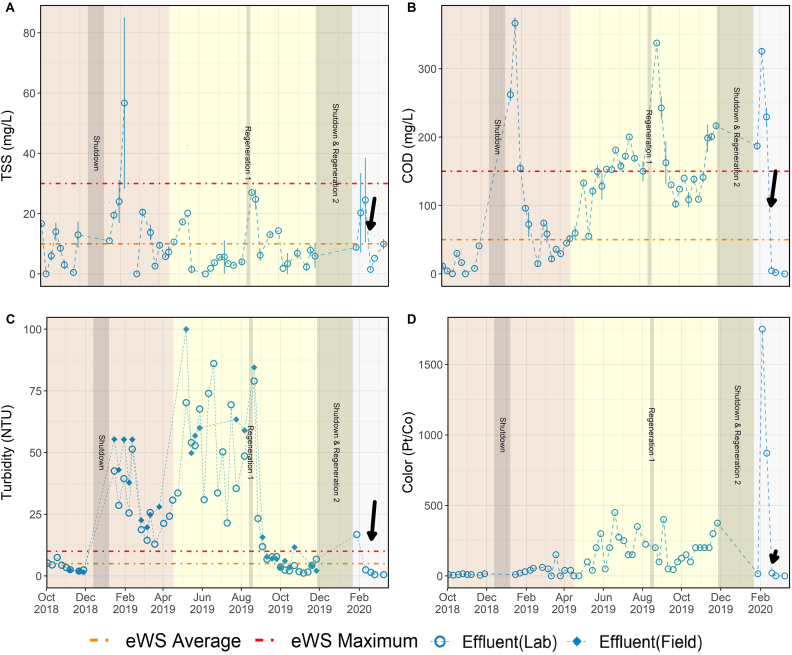
Close-up view of effluent water quality for TSS. COD, turbidity and color. The orange and red lines in Plots A, B also correspond with ISO 30500 Category A and Category B criteria, respectively. The diamond point in Plot C indicate the turbidity when the samples were taken onsite. The black arrow indicates the replacement of the GAC bed on 18 February 2020. (For interpretation of the references to color in this figure legend, the reader is referred to the Web version of this article.)

Considering the strength of the influent COD and TSS, the NG system performed well in terms of producing a relatively clean effluent low in COD and TSS. However, it did not consistently meet ISO and eWS effluent targets. It was able to meet the ISO 30500 Category B and eWS standards for TSS and COD 81.0% and 62.1% of the time, respectively. The NG effluent met the Category A standards for TSS and COD 51.7% and 27.6% of the time, respectively. The AnMBR was responsible for the bulk of COD removal, with the GAC bed in the NCS providing a critical polishing step prior to chlorination. The GAC bed was in service for 519 d (including dormancy periods) without replacement, however, a slight yellow tint could be seen in the product water after mid-2019, aligning with the addition of YW to the influent wastewater and an observed increase in color (Fig. 6D). When the GAC bed was replaced on 18 February 2020, it was evident that the exhaustion of the GAC bed was responsible for the gradual increase of COD (Fig. 6B) and color in the post-NCS samples, forcing the EC to operate for longer durations to reach the designated ORP setpoint. A significant improvement of the final product water quality was immediately apparent after the GAC replacement. The parameter values before and after GAC bed replacement were as follows: 10.6 ± 10.4 mg/L before and 5.5 ± 4.2 mg/L after for TSS, 124 ± 89 mg/L before and 2.2 ± 2.3 mg/L after for COD, 37.8 ± 94.7 NTU before and 0.8 ± 0.5 NTU after for turbidity, and 164 ± 272 Pt/Co before and 6.7 ± 11.5 Pt/Co after for color. Understanding the linkage across subsystems was critical in determining potential failure mechanisms and acted as a guideline for setting the maintenance requirements for NG. These findings suggest that regular replacement of the GAC bed, such as every 6 months as part of a preventive maintenance plan, would ensure consistent effluent water quality at this site.

However, determining an appropriate GAC replacement rate for all future NG systems is challenging because GAC exhaustion is a function of COD loading, which will vary by location. The use of a real-time COD monitoring system combined with flow sensors would allow the operators to identify NG's COD loading rates. Yet, in-line COD monitoring systems are expensive, generate hazardous waste, and require routine calibration and replenishment of chemicals. For NSSS, they are not viewed as a feasible option. By comparison, measuring color and turbidity are less expensive and easier to monitor. Both parameters have consumer-grade equipment options that are commercially available.

Correlation analysis was conducted to determine whether color or turbidity measurements would serve as an adequate surrogate for COD monitoring (further discussed in Supplemental Materials). COD showed a stronger positive correlation with color than with turbidity (Fig. S12), indicating that color is a better surrogate for proxy measurement of residual COD. These findings suggest that color, if monitored through an inline sensor, can be used as a real-time indicator of the saturation level of the GAC bed and can indicate when replacement is necessary. The increased concentration of COD observed in the effluent after prolonged dormancy should be preemptively mitigated to reduce the risk of exceeding the ISO standard. This may be accomplished through operational controls including reducing flow rates during restart, increasing chlorination run times, or replacing the GAC bed after prolonged dormancy periods. Reduction in flow rate during system restart can help ensure longer contact times in the GAC bed. Another strategy is to recirculate the effluent through the system prior to discharge to restabilize the water quality before the effluent is either reused or discharged to the environment.

### 4.2 Additional lessons learned

The system demonstrated ISO 30500 certification readiness by meeting almost all ISO and eWS standards for discharge and reuse. The field trial revealed that onsite monitoring of sub-systems performance is critical to ensure the overall reliability and performance over years of use. The AnMBR step was particularly effectively at removing the majority of the TSS, COD, and turbidity from the influent. The maintenance requirements of the membrane are low, with only an annual chemical cleaning anticipated. Furthermore, the AnMBR has sufficient capacity to handle higher OLRs than what was observed during this study.

Incorporating the NCS to the updated version of the NG system ensured that the ISO requirement for TN removal was met a majority of the time and only fell under the standard requirements when the zeolite beds became saturated. Monitoring the saturation status of the NCS is essential in ensuring that consistent nitrogen removal occurs. The field trial showed that the NCS was successful at removing TN before exhaustion and that it was possible to recover the capacity through multiple onsite regenerations. The regeneration process used NaCl and NaOH, which are common chemicals easily available worldwide. Additional work will be needed to optimize the regeneration procedure to decrease the use of materials and shorten the system down time.

Meeting the ISO 30500 TP requirements remains the greatest challenge. Modifications to the NCS can be accommodated to improve TP removal, however, further research is still required to identify the most effective technological approach to reliably remove TP at a low cost and with minimal maintenance.

During Stage B, it was clear that the addition of urinal water increased the TN loading and pushed the NCS to exhaustion. For future applications of the NG system where the front-end toilets/urinals can be designed as part of an integrated sanitation platform, urine diversion should be prioritized. Source separation of urine and upstream resource recovery would reduce the TN loading to the NCS, thereby reducing the frequency of onsite regeneration and the associated system down time, labor, and cost.

In terms of pathogens and microbial indicators, *E. coli* was tested at each major stage of the system and met the ISO 30500 standard. In all the tested final product water samples, *E. coli* was not detectable and reached the required ISO standard of 6 LRV. Samples were analyzed for *Ascaris* every month to ensure that the system adequately removed helminths so that the effluent could be safely reused. While a small number of helminths were detected in influent, no viable ova were found in the effluent. Although the system had high removal efficiencies, a few operational changes can be made to improve the system's performance. Periodic tank cleaning or chlorine dosing to the lines and tanks located after the membrane will help to reduce the risk of any potential bacterial regrowth. Additionally, due to the potential concentration of pathogens and helminths in the anaerobic sludge, thermal or chemical treatment of the sludge will be necessary as part of annual or biannual desludging to render the pathogens inactivated.

## 5 Conclusion

The South African context provided a new challenge to the NG technology with high-strength WW. The NG system was able to treat the WW and produce high quality effluent. The field trial of the NG system in the eThekwini Municipality revealed the system's strengths as well as areas requiring additional improvement to consistently meet the entirety of the ISO 30500 standard. Many of the outstanding critical parameters, such as TP, could likely be achieved with the next modified version of the NG system using the invaluable knowledge gained from the EFT. The NCS proved to be a critical subsystem. The periodic regeneration of the zeolite bed and the timely replacement of the GAC bed are key activities of scheduled preventive maintenance. The field trial highlighted room for operational improvements and identified key focal areas for continued research and development. Future work will include operating the system in a closed-loop mode to provide recycled water to the CAB for toilet flushing. The potential build-up of constituents over extended periods, and the possible impacts on user experience or treatment processes, will be examined.

## Author contribution

**Hsiang-Yang Shyu:** Conceptualization, investigation, methodology, data curation, formal analysis, writing - original draft, writing - review & editing. **Robert Bair:** Conceptualization, project administration, investigation, methodology, data curation, formal analysis, writing - original draft, writing - review & editing. **Cynthia Castro:** Conceptualization, project administration, investigation, methodology, data curation, formal analysis, writing - original draft, writing - review & editing. **Lindelani Xaba:** Investigation, resources, methodology, data curation. **Manuel Delgado-Navarro:** Conceptualization, investigation, methodology. **Rebecca Sindall:** Investigation, project administration, resources, supervision, writing – review & editing. **Ruth Cottingham:** Investigation, project administration, resources, writing – review & editing. **A. Erkan Uman:** Investigation, methodology, writing – review & editing. **Christopher A. Buckley:** Conceptualization, investigation, resources, funding acquisition, supervision. **Daniel Yeh:** Conceptualization, investigation, methodology, project administration, funding acquisition, supervision, writing - original draft, writing – review & editing. **H.Y.S**., **R.A.B**., and **C.J.C**. all contributed equally to this study.

## Declaration of competing interest

The authors declare the following financial interests/personal relationships which may be considered as potential competing interests: Daniel Yeh and Robert Bair are named inventors on patent applications for technologies related to the NEWgenerator. USF is the assignee on the patents, and has licensed technologies related to the NEWgenerator to companies in India and South Africa. Daniel Yeh and Robert Bair are co-founders of BioReNEW, inc.
