# Supplementary material for: The NEWgeneratorTM non-sewered sanitation system: Long-term field testing at an informal settlement community in eThekwini municipality, South Africa
Source: J Environ Manage. 2021 Oct 15;296:112921. doi: 10.1016/j.jenvman.2021.112921 (PMC8404038; doi:10.1016/j.jenvman.2021.112921)
Supplement: Multimedia component 1 [file mmc1.docx]

The NEWgenerator^TM^ Non-Sewered Sanitation System: Engineering Field Testing in eThekwini Municipality, South Africa

Hsiang-Yang Shyu^1^, Robert A. Bair^1^, Cynthia J. Castro^1^, Lindelani Xaba^2^, Manuel Delgado-Navarro^1^, Rebecca Sindall^2^, Ruth Cottingham^3^, A. Erkan Uman^1^, & Daniel H. Yeh^1*^

**Supplementary Materials**

**Electrochlorination (EC) system**


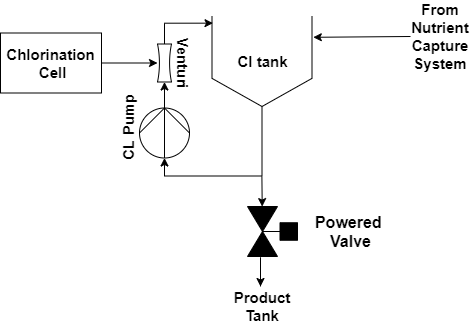


*Figure S1. Detailed schematic for the electrochlorination (EC) system utilized in the NG.*

The EC system used within the NG is a chloroalkaline process that produces chlorine gas and hydrogen from a salt solution (NaCl). The chlorination cell (M100, WaterStep, Louisville, KY, USA) is a two-chamber electrolytic cell that utilizes an ion exchange membrane to regulate the transfer of ions between two chambers, one containing an anode and the other a cathode. Chlorine gas is produced in the anode chamber, which contains the salt solution. A recirculation loop external to a chlorine contact tank draws the chlorine gas into the tank via a venturi tee, as detailed in Fig. S1. Once in solution, the chlorine gas hydrolyzes to become hypochlorous acid (HOCl). Although the primary function of chlorination dosing is for disinfection, the chlorine also serves to remove residual organics or ammonium (through breakpoint chlorination). It is also known that chlorine can oxidize inorganics such as soluble iron, magnesium, and hydrogen sulfide into solids that readily precipitate out of solution. Some of these chemical reactions are described in the following three equations (adapted from Black and Veatch, 2009):

$$2{Fe}^{2+}+{Cl}_{2}+6H_{2}O \to2Fe{(OH)}_{3\left( s \right)}\downarrow+2Cl^{-}+6H^{+}$$

$${Mn}^{2+}+ {Cl}_{2}+{2H}_{2}O \to{MnO}_{2\left( s \right)}\downarrow+ {4H}^{+}+ {2Cl}^{-}$$

$$HOCl+ H_{2}S \to S^{0}\downarrow+ H^{+}+ {Cl}^{-}+H_{2}O$$

It is likely that one of these, or a similar chemical reaction, caused the increase of turbidity observed in the final product water during portions of the field trial (see Fig. S12). Sulfur containing waters are particularly susceptible to rapid turbidity formation during chlorination (Black & Veatch, 2009) Observations from the field indicated a thin layer of white and yellowish precipitation in the final product tank, which was likely an inorganic precipitant.

**System and site photos**


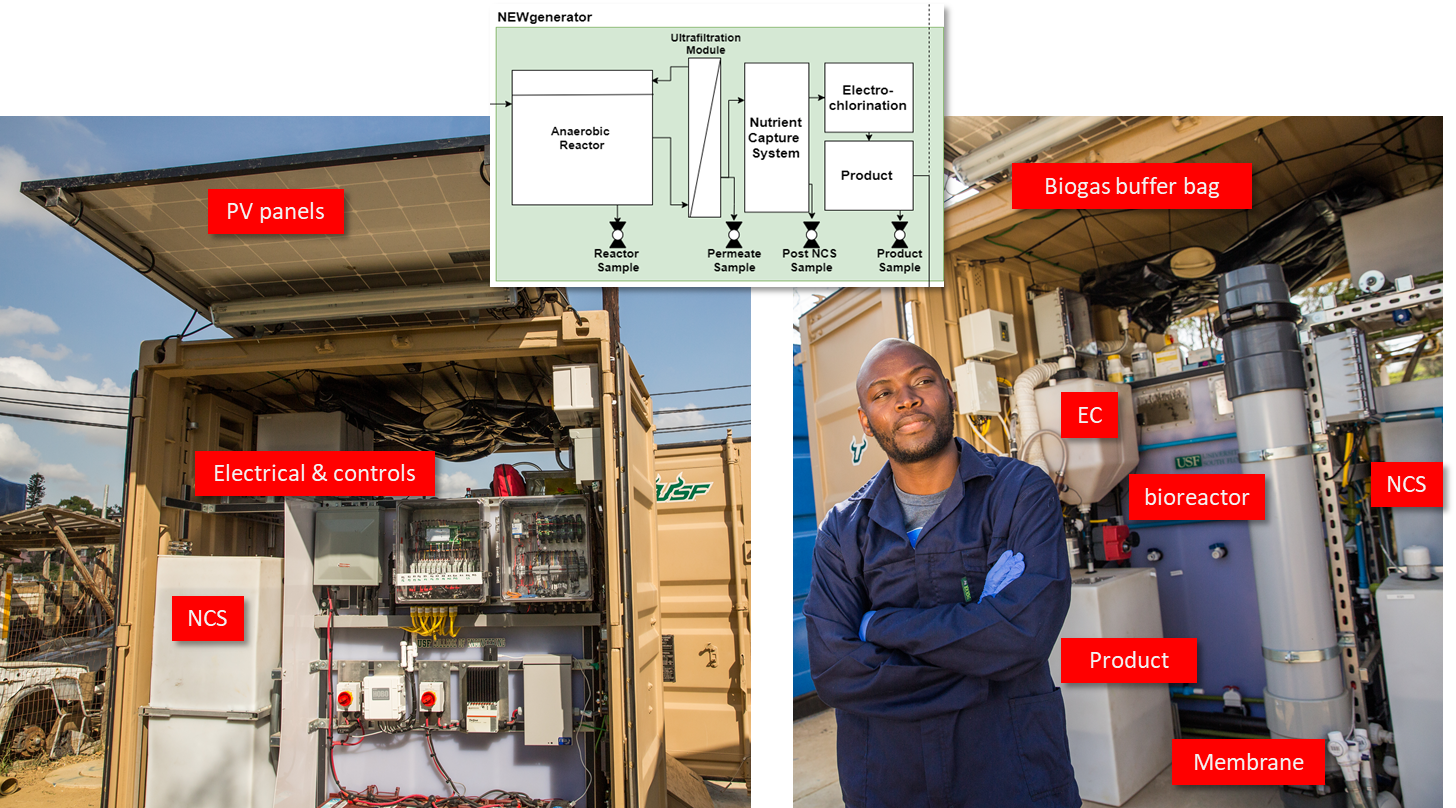


*Figure S2. Photo of the NEWgenerator and its onsite prototype engineer, Lindelani Xaba, with major subsystems and components indicated.*

*
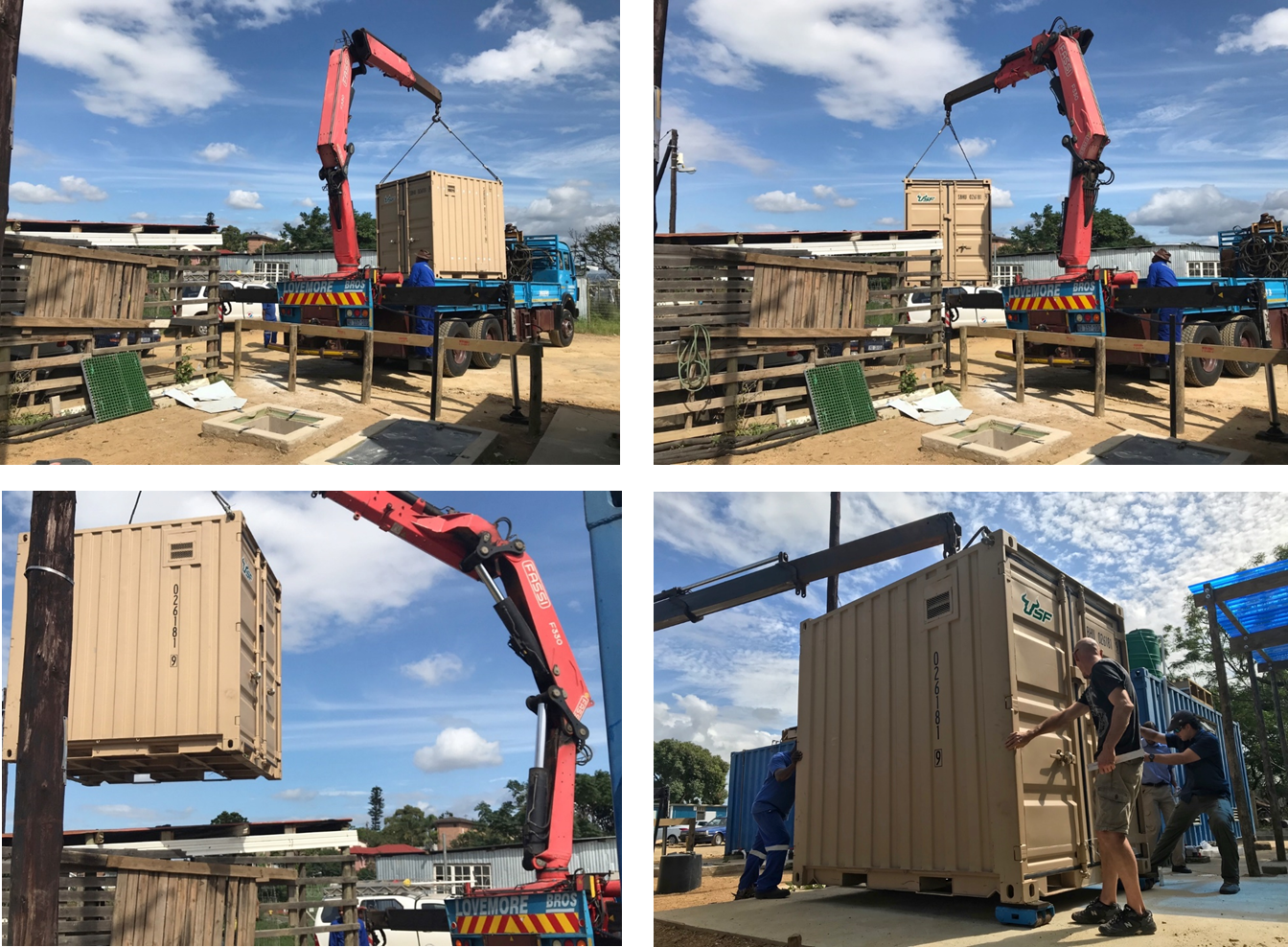
*

*Figure S3. Transportation and installation of NEWgenerator on the EFT site.*

*
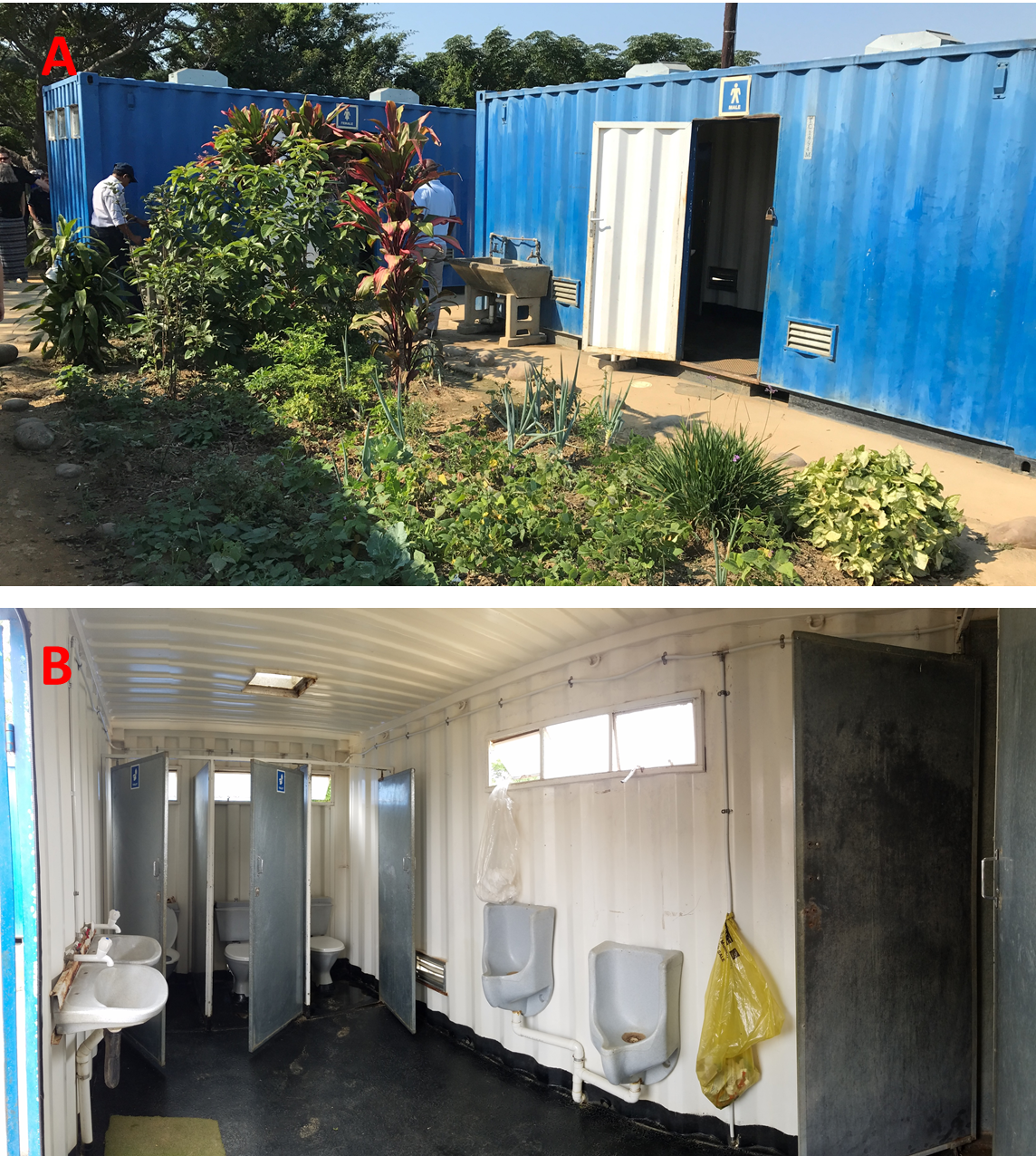
*

*Figure S4.Community ablution blocks (CABs) in the informal settlement community: (A) the exterior of the male and female CABs, along with a community garden; (B) the interior of the male CAB used for this study.*

*
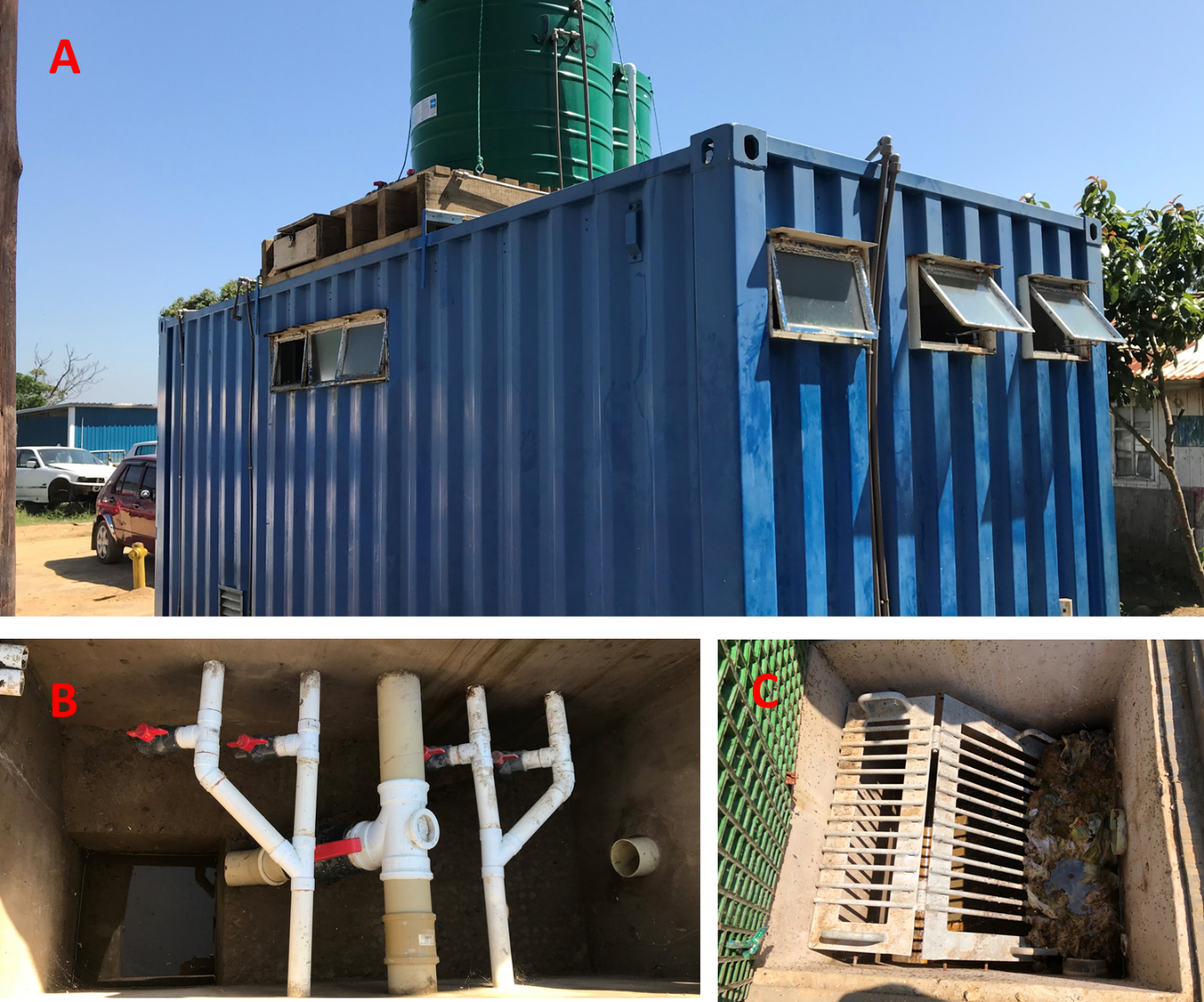
*

*Figure S5.Engineering field test (EFT) site: (A) exterior of the male CAB; (B) valving chamber to isolate wastewater source that enters the NEWgenerator; and (C) screen pit, where trash is screened and removed.*


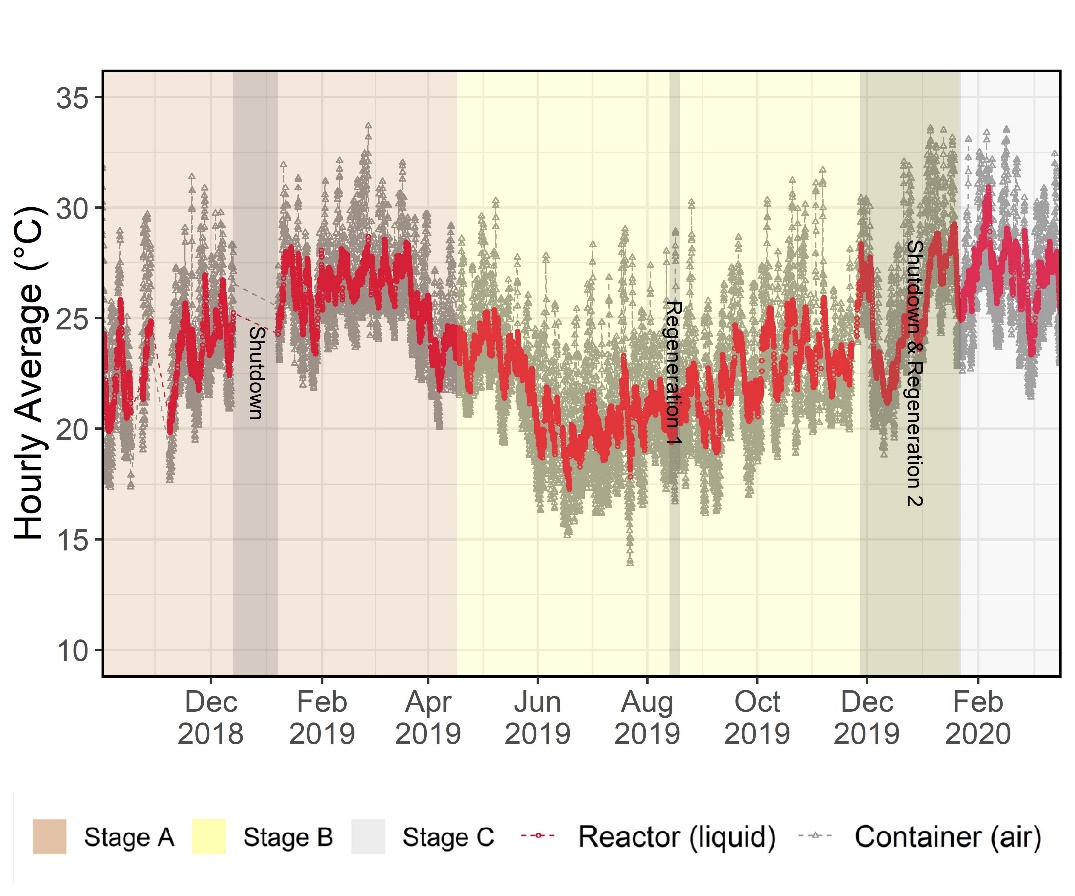


*Figure S6. Hourly ambient temperature inside the NG container (air) and bioreactor (liquid) over the three testing stages.*

During the entire testing period, the NG system was operated under ambient temperature (ranging from 13.8${}^{\circ}$C to 34.0${}^{\circ}$C) without any temperature control (Fig. S6). In addition to seasonal temperature changes, the unit also experienced daily temperature swings. The range of daily temperature fluctuation was less within the reactor (ΔT <3°C) due to dampening from the thermal capacity of the liquid, compared to the ambient air in the container (ΔT up to 15°C).

During Stage A, the average reactor temperature was 25.0 ± 2.0 ${}^{\circ}$C, (28.7 ${}^{\circ}$C max, 19.8 ${}^{\circ}$C min). During Stage B, the average reactor temperature was 22.6 ± 2.3 ${}^{\circ}$C (29.3 ${}^{\circ}$C max, 17.3${}^{\circ}$C min). During Stage C, the average reactor temperature was 27.1 ± 1.4 ${}^{\circ}$C (30.9${}^{\circ}$C max, 23.4${}^{\circ}$C min).

## Influent characterization

*
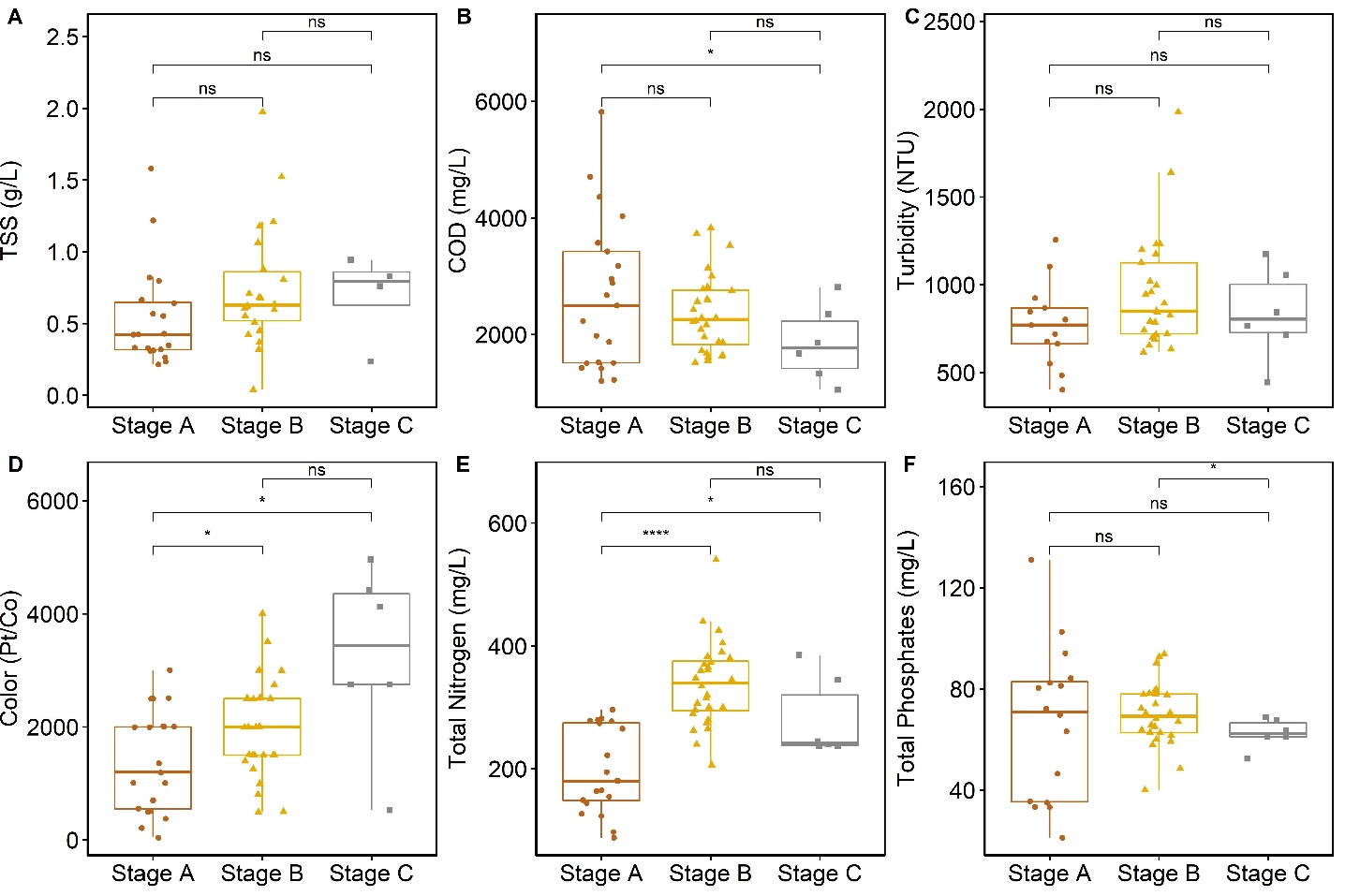
*

*Figure S7. Influent characteristics broken down into their corresponding stages. Lines indicate medians, boxes 25^th^ and 75^th^ percentiles. One-way ANOVA with a Sidak’s multiple comparisons test was used to compare measurements between each stage of influent characteristic. ‘ns’ = P > 0.05, ‘*’ = P = 0.05, ‘**’ = P = 0.01, ‘***’ = P = 0.001, ‘****’ = P = 0.0001. Statistical analysis was performed with R.*

The following observations can be made on the influent characteristics (Fig. S7): 1) A seemingly gradual increase of TSS from Stage A to Stage B to Stage C, which was not statistically significant. 2) An increase of color from Stage A to Stage B to Stage C, which was statistically significant. The increase in color through the stages was puzzling as there was no statistically significant change in COD or turbidity from Stage A to B to C. 3) A strongly significant (P=0.0001) increase (roughly doubling) of TN from Stage A to Stage B due to the addition of yellow water (YW), then a slight decrease from Stage B to Stage C with the removal of YW. Comparatively, there was no difference in TP with the inclusion or exclusion of YW.

*
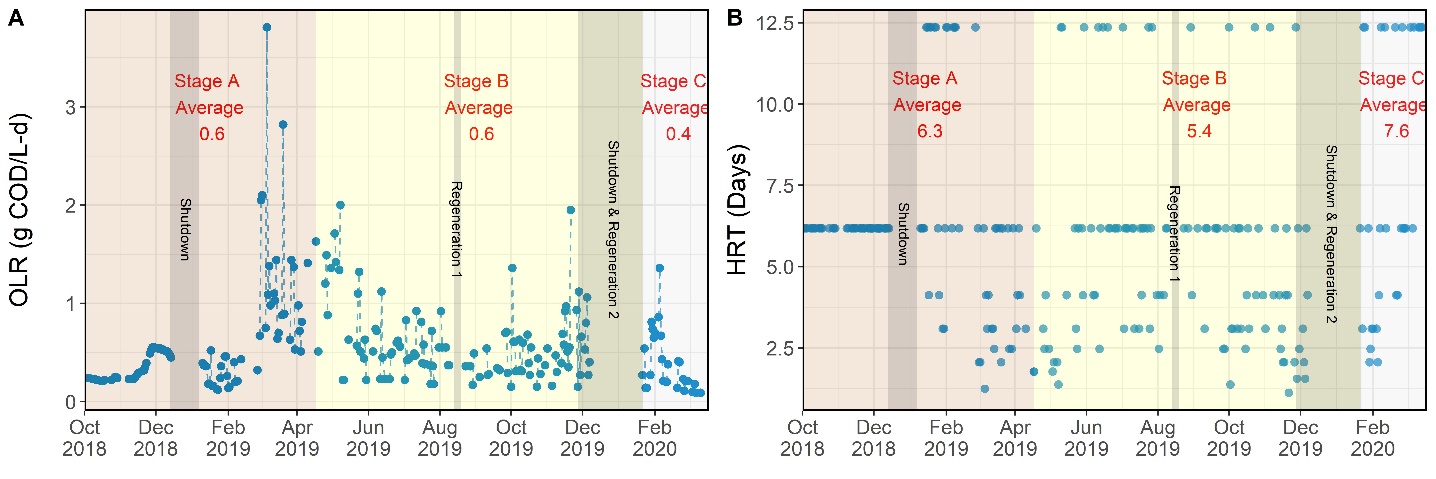
*

*Figure S8. OLR and HRT over the three testing stages. The red number show the average of the OLR for each stage.*

The organic loading rate (OLR) and hydraulic retention time (HRT) were calculated with the following equations:

$OLR=\frac{{Q C}_{0}}{V}=\frac{C_{0}}{HRT}$ $HRT=\frac{V}{Q}$

where OLR = organic loading rate (g-COD/L-d); $C_{0}$= influent substrate concentration (g-COD/L); Q = influent flow rate (L/d); V = AnMBR reactor volume (L); HRT = hydraulic retention time (d) of the AnMBR.


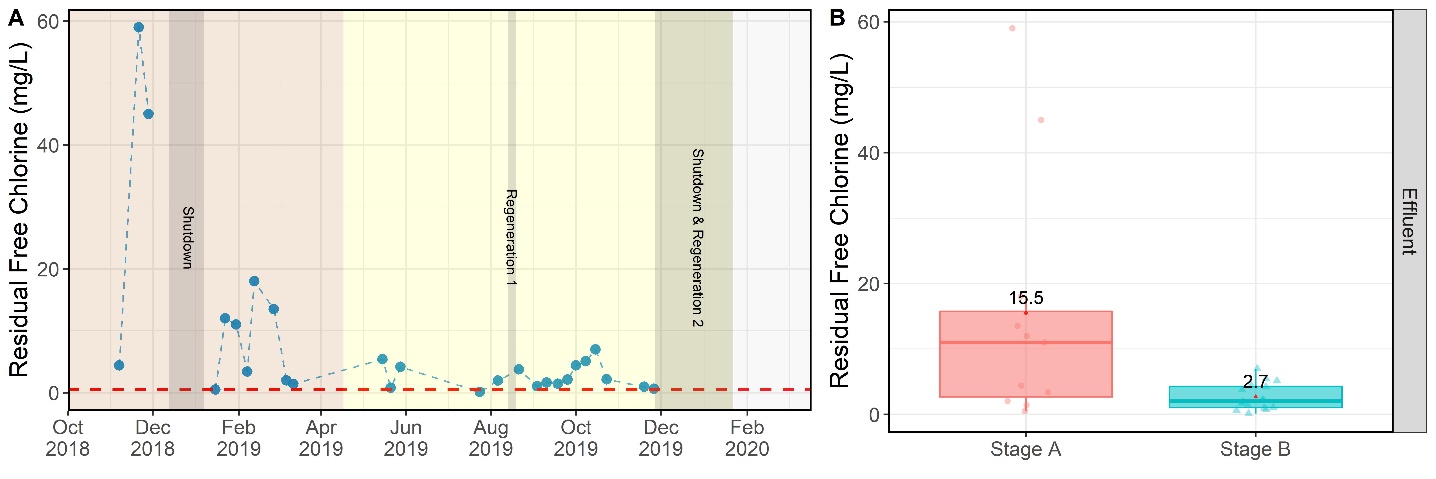


*Figure S9. Free chlorine residual in the final product water. Plot A show the effluent concentrations, with dotted lines indicating eWS standard. The lines in the box indicate medians, the red dots and the number shown in the box is the average for each parameter, boxes 25^th^ and 75^th^ percentiles. The first grey bar shows the timeframe of first shutdown period. Second grey bar shows the timeframe of regeneration of the NCS.*


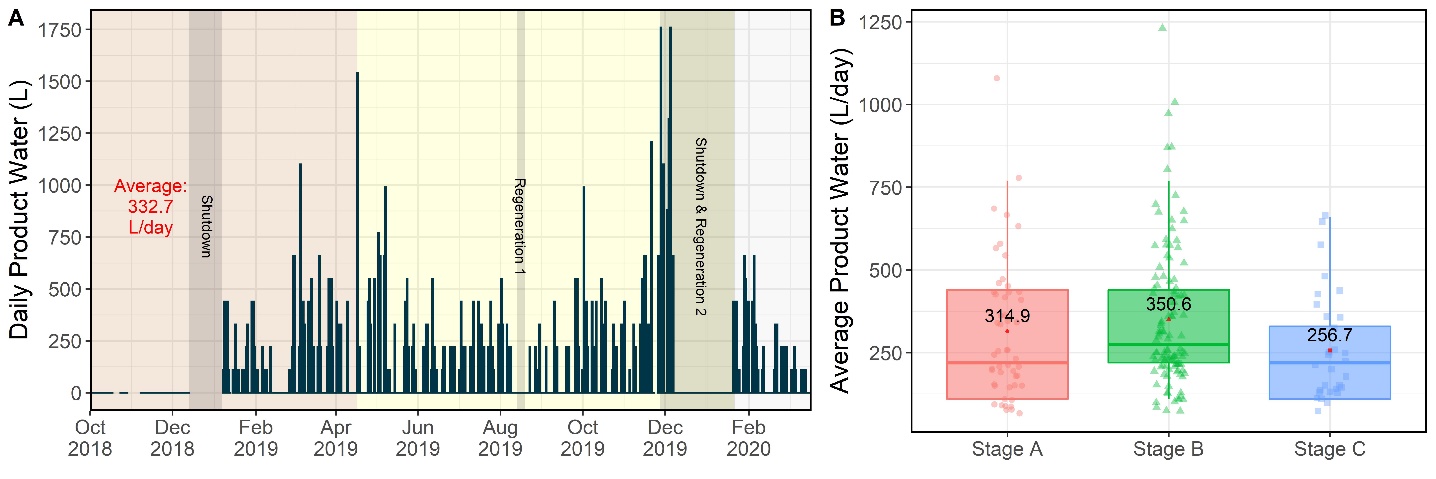

*Figure S10. Product water throughput (L/d) over the three testing stages. The average daily product water volume does not include days the system was not operated. Plot A show the daily water production volume. Plot B shows the boxplot of each water production in different stage of the field test. The initial flow data (2018) was lost due to sensor malfunction.*

*Figure S11. Membrane performance as indicated by transmembrane pressure (TMP). Graph shows the averaging hourly TMP. The red dotted lines indicating the two membrane-cleaning events.*

*
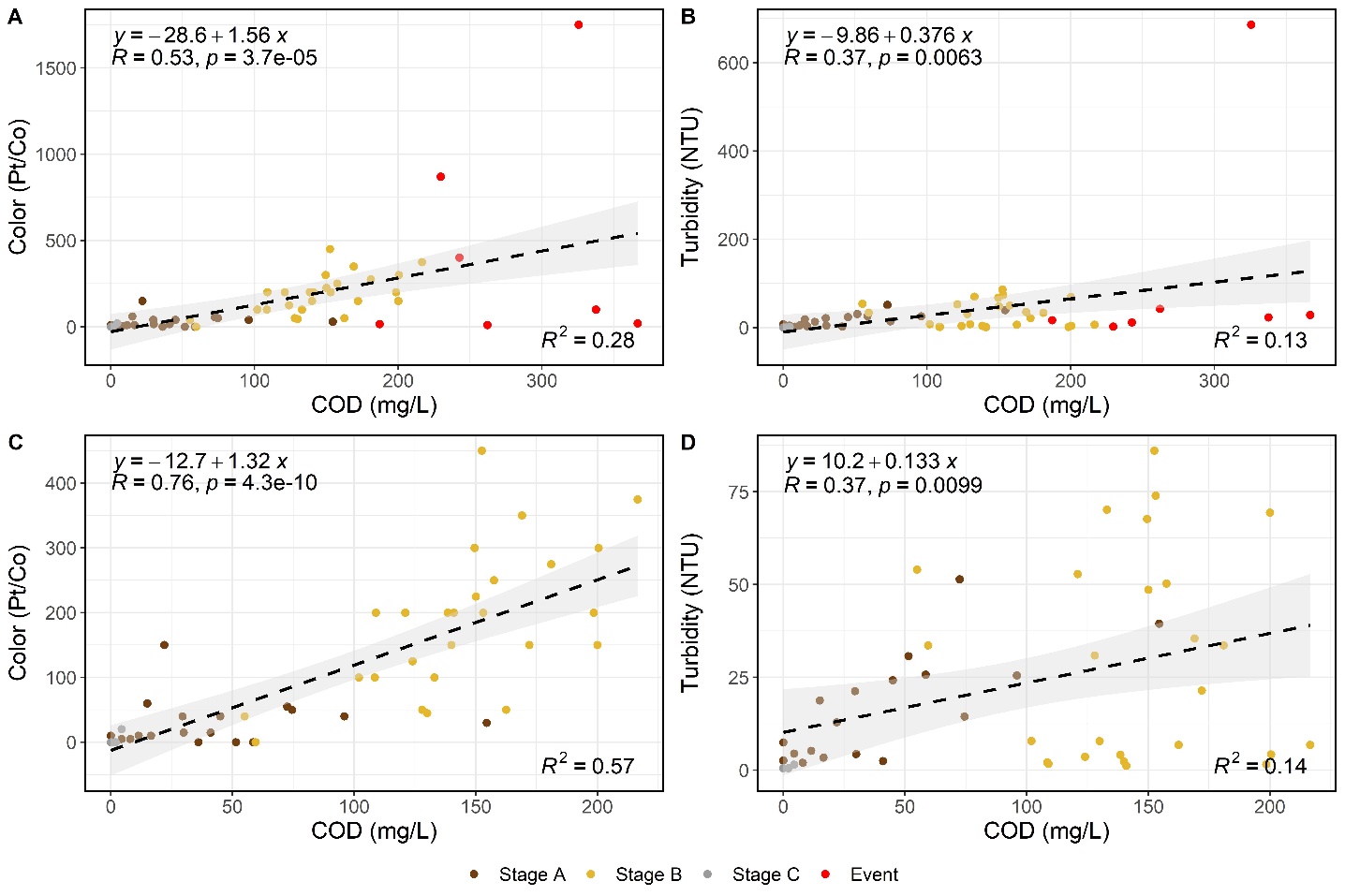
*

*Figure S12. Effluent COD correlation between color and turbidity. Plots A and B show the complete data set, while C and D exclude data immediate after a restart. The data removed include the sample immediately after NCB regeneration (27 Aug, 2019) and two shutdown periods (15 Jan, 2019 and 28 Jan, 2020). R is the correlation coefficient; p (or p-value) is the significance level of the correlation.*

A Pearson’s correlation test was used to determine if any linear correlation existed between effluent COD and potential surrogate parameters such as color and turbidity (Fig. S12). Although not a perfect surrogate, color was more positively correlated to COD than turbidity, with an R value of 0.53 and 0.37 using raw data. This may be due to the fact that turbidity also includes occasional inorganic precipitates generated by oxidation in EC, which are not part of COD. Additionally, it was observed that the restart data obtained immediately after a long-term shutdown did not follow the same trend as the data obtained during normal operation. Immediately after these events, the ratio between COD and color would shift. This effect was not as pronounced with the relationship of COD and turbidity. Once these restart data points were omitted, the correlation between COD and color became even stronger, with an R value of 0.76.

**References**

Black & Veatch Corporation, 2009. White’s Handbook of Chlorination and Alternative Disinfectants, 5th Edition. John Wiley & Sons, Inc.
